# Blocking aryl hydrocarbon receptor remodels myeloid–T cell crosstalk and enhances anti-PD-1 response in the tumor microenvironment

**DOI:** 10.1186/s40164-026-00843-8

**Published:** 2026-10-26

**Authors:** Dong Kwon Kim, Sujeong Baek, Youngtaek Kim, Seung Min Yang, So Young Park, Su Jin Choi, Ju Hyeon Lee, Sang Hoon Lee, Jongho Cho, Daewon Cha, Dajeong Kim, Sunghwa Kim, Jae Hwan Kim, Byoung Chul Cho

**Affiliations:** 1 Severance Biomedical Science Institute, Yonsei University College of Medicine, Seoul, South Korea; 2 Brain Korea 21 PLUS Project for Medical Science, Yonsei University College of Medicine, Seoul, Republic of Korea; 3 Yonsei New Il Han Institute for Integrative Lung Cancer Research, Yonsei University College of Medicine, Seoul, Korea; 4 Dong-A ST Research Institute, Yongin-si, Korea; 5 Division of Medical Oncology, Department of Internal Medicine, Severance Hospital, Yonsei Cancer Center, Yonsei University College of Medicine, Seoul, Korea

**Keywords:** Aryl hydrocarbon receptor, Immunotherapy, Maintenance chemotherapy, Neoadjuvant therapy, Immune checkpoint inhibitor

## Abstract

**Purpose:**

Cancer metabolism-associated immune suppression remains a major barrier to durable therapeutic responses. Indoleamine-2,3-dioxygenase 1 has been pursued as a therapeutic target, but incomplete pathway suppression has limited clinical success. As the aryl hydrocarbon receptor is a ligand-activated transcription factor and downstream effector of tryptophan metabolism, we investigated how myeloid-cell aryl hydrocarbon receptor signaling shapes the tumor immune microenvironment and whether its inhibition can restore antitumor immunity.

**Methods:**

Conditional aryl hydrocarbon receptor knockout mice targeting dendritic cells or macrophages were used to evaluate the effects of genetic aryl hydrocarbon receptor ablation. Pharmacologic inhibition was assessed using DA-4505 across syngeneic lung, colorectal, and breast cancer models and an immune checkpoint inhibitor–refractory humanized patient-derived xenograft model. Therapeutic efficacy was evaluated alone or in combination with anti–programmed cell death protein 1 therapy, chemotherapy, or surgery. Tumor growth, survival, metastasis, and immune remodeling were analyzed by flow cytometry, multiplex immunohistochemistry, single-cell RNA sequencing, cytokine profiling, and human tumor datasets.

**Results:**

Conditional aryl hydrocarbon receptor depletion in macrophages and dendritic cells enhanced innate and adaptive immune activation, increased interferon-γ–producing T cells, and strengthened antitumor immunity. In mouse tumor models, pharmacologic aryl hydrocarbon receptor blockade enhanced macrophage chemotaxis and activation programs, promoted M1-like macrophage polarization, increased interferon-γ–producing CD8⁺ T cells, and reduced regulatory T cells and polymorphonuclear myeloid-derived suppressor cells. These changes promoted robust T-cell activation and durable tumor control. Aryl hydrocarbon receptor inhibition further synergized with chemotherapy, surgery, and immune checkpoint blockade, leading to expansion of effector and memory T-cell populations and sustained antitumor responses. In a humanized lung cancer model, aryl hydrocarbon receptor blockade increased intratumoral CD8⁺ T-cell infiltration and cytotoxic-memory programs while reducing regulatory T-cell enrichment. In human tumor datasets, aryl hydrocarbon receptor–positive macrophages correlated with FOXP3⁺ T-cell infiltration, and higher aryl hydrocarbon receptor inhibition-associated T-cell signatures were associated with improved patient survival.

**Supplementary Information:**

The online version contains supplementary material available at https://doi.org/10.1186/s40164-026-00843-8.

## Introduction

Cancer immunotherapies have become central components of modern cancer treatment and are increasingly combined with chemotherapy, surgery, and radiotherapy [1]. However, many patients fail to achieve durable clinical benefit, particularly those with immunologically “cold” tumors or highly immunosuppressive tumor microenvironments (TME) [2]. Therefore, therapeutic strategies that can reprogram the TME and enhance antitumor immune responses remain urgently needed [3, 4].

Tryptophan metabolism has begun to attract considerable attention in cancer research. Indoleamine 2,3-dioxygenase 1 (IDO1) is a heme-containing enzyme that catalyzes L-tryptophan oxidation and plays a crucial role in cancer immune evasion [5, 6]. Accordingly, IDO1 inhibitors have been explored as therapeutic agents, with compounds such as epacadostat, BMS-986,205, and PF-06840003 undergoing preclinical validation and clinical trials [7–9]. However, the phase III clinical trial (ECHO-301) evaluating a combined epacadostat and immunotherapy treatment failed to demonstrate significant clinical benefits, leading to the suspension or discontinuation of most IDO1 inhibitor trials [7]. These outcomes suggest that targeting IDO1 alone may be insufficient, in part because KYN production and downstream immunosuppressive signaling can persist through alternative metabolic pathways [10–14].

Given these limitations, the aryl hydrocarbon receptor (AhR) has emerged as a promising alternative therapeutic target. IDO1 catalyzes the oxidative degradation of tryptophan into kynurenine (KYN), an endogenous AhR ligand [15–18]. Upon ligand binding, AhR translocates to the nucleus and functions as a ligand-activated transcription factor that regulates genes involved in immune modulation, metabolism, and cell differentiation. Thus, direct AhR inhibition may provide a strategy to suppress downstream immunoregulatory signaling that can persist despite incomplete blockade of KYN production [19].

In cancer, activation of the IDO1-KYN-AhR axis has been implicated in the establishment of immunosuppressive TME. AhR signaling promotes regulatory T-cell (Treg) differentiation and suppressive function through transcriptional programs associated with FoxP3 stability and immunoregulatory cytokines, including IL-10 and TGF-β [20–23]. In the myeloid compartment, AhR activation has been linked to tumor-associated macrophage polarization and the accumulation or functional maturation of suppressive myeloid populations, including M-MDSCs and PMN-MDSCs. Through these coordinated effects on adaptive and innate immune compartments, AhR signaling may limit cytotoxic CD8⁺ T-cell activity and contribute to resistance to immunotherapy. Despite growing evidence linking AhR to tumor immune suppression, the mechanisms by which AhR blockade reshapes the TME remain incompletely understood. In particular, how AhR inhibition alters myeloid–T-cell crosstalk, systemic immune activation, and responses to immune checkpoint blockade or chemotherapy have not been fully defined. Addressing these unresolved mechanisms may provide a rationale for investigating AhR inhibition as a therapeutic strategy to restore antitumor immunity and enhance the efficacy of existing cancer treatments [24–27].

In addition to its role in shaping an immunosuppressive TME, AhR signaling has been associated with responses to chemotherapy and immunotherapy, prompting efforts to enhance antitumor efficacy through AhR blockade [28]. However, the mechanisms by which AhR inhibition reshapes therapy-induced immune responses remain incompletely understood. In particular, how AhR blockade modulates myeloid cell function, T-cell activation, and myeloid–T-cell crosstalk within the TME, and how these changes influence responses to immune checkpoint blockade or chemotherapy, requires further investigation. Addressing these questions may provide a rationale for combining AhR inhibitors with established cancer therapies to overcome immune suppression and improve durable antitumor responses [29, 30].

In this study, we investigated how AhR inhibition reprograms systemic and local antitumor immunity. Using genetic and pharmacologic approaches across multiple tumor models, including immune checkpoint inhibitor–resistant settings, we examined the effects of AhR blockade on myeloid cell function, T-cell activation and differentiation, and therapeutic responses to immune checkpoint blockade, chemotherapy, and surgery^29^. For pharmacologic AhR blockade, we used DA-4505, a novel AhR inhibitor selected for in vivo therapeutic assessment based on the potent AhR inhibitory activity, favorable selectivity profile, and minimal direct cytotoxicity of its active form, SB3181. Our findings define AhR as a regulator of myeloid–T-cell crosstalk and provide a rationale for combining AhR inhibition with established cancer therapies.

## Methods

### Cell lines and reagents

Human A549 (CCL-185) and H2228 (CRL-5935) cell lines were purchased from the American Type Culture Collection (ATCC; Manassas, USA), and the HepG2 (KCLB No. 88065) cell line was purchased from the Korean Cell Line Bank (KCLB; Seoul, Korea).

Mouse TC-1 (CRL-2785, RRID: CVCL_4699), LLC1 (CRL-1652, RRID: CVCL_4358), CT26 (CRL-2638, RRID), and 4T1 (CRL-2539) cell lines were purchased from ATCC and the Hepa1c1c7 (KCLB No. 22026) cell line was purchased from KCLB. The A549, H2228, TC-1, CT26, and 4T1 cell lines were cultured in RPMI-1640 medium (Cat.SH30027.01, Cytiva, Washington, USA). The LLC1 cell line was cultured in DMEM (#10-013-CV, Corning, Tewksbury, USA), and HepG2 and Hepa1c1c7 cell lines were cultured in MEM (#10-010-CV, Corning), respectively, supplemented with 10% FBS (Cat. 12483020, ThermoFisher Scientific; Waltham, USA) and 1% antibiotic-antimycotic (Cat. 15240096, ThermoFisher) in a humidified incubator with 5% CO_2_. All cell lines were purchased from authenticated sources (ATCC or KCLB). Mycoplasma contamination was tested using a detection kit (SMD0172), and all results were negative. All cell lines were used within 15 passages and maintained for less than 6 months. All reference compounds were purchased from Selleck Chemicals (Houston, USA, RRID: SCR_003823). The AhR inhibitor DA-4505 was provided by Dong-A ST Inc. SB3181, the prodrug version of DA-4505, was used for the in vitro assay.

### Mice

CD11c-cre (008068, RRID: IMSR_JAX:008068) and LysM-cre (004781, RRID: IMSR_JAX:004781) mice were purchased from The Jackson Laboratory (Bar Harbor, USA). CRISPR LoxP knock-in was used to generate AhR^LoxP^ mice. LysM^Cre^-AhR^LoxP^ and CD11c^Cre^-AhR^LoxP^ were derived in our lab by crossing CRISPR AhR^LoxP/LoxP^ C57BL/6 mice. Mice with specific AhR deletions in macrophages or dendritic cells were generated by crossing LysM^Cre^ mice with AhR^LoxP/LoxP^ and CD11c^Cre^ mice with AhR^LoxP/LoxP^, respectively. AhR deletion in macrophages or dendritic cells was verified by PCR (Supplementary Fig. S1). Humanized mice used in our study were purchased from The Jackson Laboratory. The patient-derived xenograft model was established as previously described [31]. Ten-week-old NOG (NOD/Shi-scid/IL-2Rγnull and RRID: IMSR_JAX:005557) mice and six-week-old C57BL/6 (RRID: IMSR_JAX:000664) and BALB/c (RRID: IMSR_JAX:000651) mice were purchased from Orient Bio (Seongnam, South Korea). Procedures involving the care and use of animals in this study were reviewed and approved by the Institutional Animal Care and Use Committee (IACUC numbers 2022-0037 and 2022-0041) before the study. Tumor volume was measured four times a week using digital calipers and calculated using the equation “V = length × width^2^ × 0.524” (where “length” = longest diameter and “width” = shortest, perpendicular diameter) [31].

### Tumor studies

For the combination treatment strategy with anti-PD-1, 6-week-old WT mice were subcutaneously (s.c.) injected with 1 × 10⁶ CT26, 4T1, LLC1 or TC-1 tumor cells. For orthotopic model, BALB/c female mice were implanted to mammary tap pad directly with 1 × 10^5^ 4T1 cells. When tumor size reached 100 mm³, mice were treated with DA-4505 (10 mg/kg, orally, once daily), anti-PD-1 (10 mg/kg, intraperitoneally (i.p.), twice a week; Bio X Cell, BE0146), or a combination of DA-4505 and anti-PD-1. In the case of humanized mouse model, pembrolizumab was injected intraperitoneally once a five days with 10 mg/kg instead of mouse anti-PD-1. Tumors were harvested at end-of the time points to analyze tumor-infiltrating immune cells.

For the knockout mouse validation, 6-week-old LysM^Cre^-AhR^LoxP^ and CD11c^Cre^-AhR^LoxP^ mice were s.c. injected with 1 × 10⁶ LLC1 or TC-1 tumor cells. Spleen and tumors were harvested at the end-of time points to analyze immune cells.

For the combination strategy with chemotherapy, 6-week-old WT syngeneic mice were s.c. injected with 1 × 10⁶ TC-1 tumor cells and then treated with DA-4505, anti-PD-1, cisplatin (5 mg/kg, i.p., twice a week) and pemetrexed (100 mg/kg, i.p., twice a week) injection initiated when tumor size reached approximately 100 mm^3^. Tumors were harvested at different time points to analyze tumor-infiltrating immune cells.

For the combination strategy with surgery, 6-week-old WT BALB/c mice were s.c. injected with 1 × 10⁵ 4T1 tumor cells and then treated with DA-4505, anti-PD-1, or a combination of DA-4505 and anti-PD-1. Treatment was administered either in the neoadjuvant setting prior to surgical tumor resection or in the adjuvant setting following tumor removal.

In addition, the antitumor efficacy of DA-4505 was evaluated in immunodeficient NOG mice model.

### Flow cytometry

To investigate the tumor and spleen immune landscape, tumor and spleen tissues were surgically excised from mice. The collected tissues were subjected to physical and enzymatic dissociation to obtain single-cell suspensions, which were processed for antibody staining and flow cytometric analysis. Dissociated single cells were washed with buffer (PBS containing 1% BSA, 0.01% Sodium Azide, 0.5mM EDTA) and blocked with FcR Blocking Reagent (Miltenyi Biotec #130-092-575) at 4 ℃ for 10 min.

For human cells, immunofluorescence antibodies were added for surface staining: for T cell analysis-PE/Cyanine7 anti-human CD3e (UCHT1, Biolegend #300420, RRID: AB_439781), PerCP anti-human CD4(RPA-T4, Biolegend #300528, RRID: AB_893321), Alexa Fluor 700 anti-human CD8a (OKT8, Invitrogen #56-0086-42, RRID: AB_10670753), APC/Cyanine7 anti-human CD25 (BC96, Biolegend #302614, RRID: AB_314284), Super Bright 600 anti-human CD45RO (UCHL1, Invitrogen #63-0457-42, RRID: AB_2662466), APC anti-human CD197 (G043H7, Biolegend #353214, RRID: AB_10917387), Super Bright 702 anti-human CD279 (eBioJ105, Invitrogen #67-2799-42, RRID: AB_2734945), Brilliant Violet 421 anti-human CD45 (HI30, Biolegend #304032, RRID: AB_2561357), Brilliant Violet 785 anti-human IFN-γ (4 S.B3, Biolegend #502542, RRID: AB_2563882), PE anti-human FoxP3 (PCH101, Invitrogen #12-4776-42, RRID: AB_1518782); for myeloid cell analysis-APC-eFluor780 anti-human CD14 (61D3, Invitrogen #47-0149-42, RRID: AB_1834358), Brilliant Violet 650 anti-human CD80 (B7-1)(2D10, Biolegend #305227, RRID: AB_11203545), PE/Cyanine7 anti-human CD11b (ICRF44, Biolegend #301322, RRID: AB_830644), FITC anti-human CD15 (HI98, Biolegend #301904, RRID: AB_314196), Brilliant Violet 605 anti-human CD86 (B7-2) (IT2.2, Biolegend #305430, RRID: AB_2563824), Super Bright 702 anti-human HLA-DR (LN3, Invitrogen #67-9956-42, RRID: AB_2717187), Super Bright 780 anti-human CD11c (3.9, Invitrogen #78-0116-42, RRID: AB_2744902), PE anti-human CD68 (Y1/82A, Biolegend #333808, RRID: AB_1089056), Brilliant Violet 421 anti-human CD45(HI30, Biolegend #304032, RRID: AB_2561357), and PerCP-eFluor 710 anti-human CD206 (19.2, Invitrogen #46-2069-42, RRID: AB_10853034). In order to analyze functional marker IFN-γ, CD80, CD86, and HLA-DR, we used mean fluorescence intensity (MFI) and percentage (%) values.

For mouse cells, the T cell population was stained with anti-CD45 (103110; Biolegend), anti-CD3e (100320; Biolegend), anti-CD4 (100526; Biolegend), anti-CD8 (100750; Biolegend), anti-CD25 (67-0251-82; Invitrogen, Waltham, MA, USA), anti-CD44 (61-0441-82; Invitrogen), anti-FoxP3 (12-5773-82; eBioscience), and anti-CD197 (120108; Biolegend) antibodies. The NK cell population (CD45^+^CD3^−^CD49b^+^) was stained with anti-CD49b (108906; Biolegend) antibodies. Activation markers were stained using anti-ki67 (652413; Biolegend), anti-PD-1 (135218; Biolegend), and anti-IFN-γ (505832; Biolegend) antibodies. Dendritic cell populations (CD45^+^F4/80^−^CD11c^+^MHC I^−^) were identified using anti-H-2Db (A15443; Invitrogen), anti-CD11c (117343; Biolegend), anti-CD80 (47-4801-82; eBioscience), and anti-CD86 (104729; Biolegend) antibodies. M1 and M2 macrophages were identified using anti-F4/80 (47-4801-82; eBioscience), anti-CD206 (17-2061-80; eBioscience), anti-CD11c, anti-CD80, and anti-CD86. Myeloid-derived suppressor cells were identified using anti-CD11b (101216; Biolegend) and anti-Ly-6G (127606; Biolegend).

AhR (LS-C783005; LSBio, WA, USA) and DRAQ5 (#62251; ThermoFisher) were used for both human and mouse cells, which were fixed and permeabilized for intracellular staining using IC Fixation Buffer (Invitrogen #00-8222-49) at room temperature for 30 min. Flow cytometry analysis was performed using a BD LSR-fortessa™ X-20 instrument (BD Bioscience, Franklin Lakes, USA). FlowJo software v10 (Tree Star, Ashland, USA, RRID: SCR_008520) was used for data acquisition and analysis. Image cytometry analysis was performed using a BD FACSDiscover™ S8 instrument (BD Bioscience). Immune-cell populations were identified using gating strategies adapted from our previously published flow cytometry analysis pipeline and modified according to the antibody panels used in this study. Representative gating strategies are shown in Supplementary Fig. S2.

### Tissue microarray (TMA)

A commercially available lung cancer tissue microarray (TMA) (catalog no. LC821, TissueArray.com, Rockville, MD, USA) was analyzed. The array included formalin-fixed, paraffin-embedded (FFPE) lung carcinoma specimens and matched adjacent normal tissues with annotated clinicopathological information. In addition, FFPE tumor tissues obtained from humanized mouse models were processed and constructed into a custom TMA block using the same protocol as for the commercial array. Briefly, representative tumor regions were identified on hematoxylin and eosin–stained sections, and 1.5-mm cores were punched and arrayed into recipient paraffin blocks. Section  (4 μm) from both TMAs were deparaffinized, rehydrated, and subjected to antigen retrieval in citrate buffer (pH 6.0), followed by immunohistochemical staining.

### Multiplex immunohistochemistry (mIHC)

Tumor tissues were fixed in formalin to create paraffin-embedded blocks and slides. The slides were automatically stained with multiple colors using a BOND RX Fully Automated Research Stainer (21.2821; Leica Biosystems, Nubloch, Germany) and an Opal Polaris 7 Color IHC Detection Kit (OP-000003, Akoya Biosciences, Massachusetts). All procedures were performed according to the manufacturer’s instructions. Multi-color microscopic fluorescence images were acquired using the Vectra Automated Quantitative Pathology Imaging System (Akoya phenoimager). Multiplex staining was performed using anti-human antibodies, anti-CD8 (#NCL-L-CD8-4B11; Leica Biosystems, Massachusetts, US Biosystems), anti-PanCK (#NBP2-29429; Leica Biosystems), anti-FoxP3 (#98377; Cell Signaling Technology; Beverly (CST)), anti-PD-L1 (#SK006; DAKO), anti-CD68 (#76437; CST), anti-AhR (#LS-C783005), anti-CD4 (#ab133616; Abcam), and anti-41BB (#34594; CST). For mice, anti-CD4 (#25229, CST), and anti-F4/80 (#70076, CST) were used. Tissue sections were counterstained with Spectral DAPI (4′,6-diamidino-2-phenylindole; FP1490; Akoya Biosciences). Fluorescence images were analyzed using inForm Advanced Image Analysis Software (inForm 2.6, Akoya Biosciences). Cells and tissues were automatically segmented using a user-educated segmentation algorithm, and the tumor nest and stroma area were calculated.

### In vivo immune cell depletion

To selectively deplete immune cell subsets, depletion antibodies (400 µg) were administered intraperitoneally twice a week, beginning one day before treatment, as follows: anti-CD8 (clone 2.43, BioXCell; Lebanon, NH), anti-CD4 (clone GK1.5, BioXCell), and anti-NK1.1 (clone PK136, BioXCell). Macrophage depletion was achieved through intraperitoneal administration of 300 µg of anti-CSF1R (clone AFS98, BioXCell) on alternate days. Plasmacytoid dendrtic cells (pDC) depletion was performed with anti-mouse CD317 (500 µg, i.p., twice a week; Bio X Cell, BE0311). Mice in the control group received 400 µg of an isotype-matched IgG control antibody (BioXCell).

### Cytokine array

Serum samples obtained from humanized mice were analyzed using a Luminex assay kit according to the manufacturer’s instructions. A 14-plex cytokine panel was used to quantify the levels of CCL2, CCL4, CXCL5, Granzyme A, Granzyme B, IFN-γ, IL-1β, IL-1ra, IL-10, IL-12p70, MMP-7, PD-L1, TNF-α, and VEGF (# LXSAHM-14, R&D Systems). Only statistically significant concentration differences between the groups were reported.

### Single-cell RNA sequencing

Tumor samples were dissociated and prepared using a single-cell library preparation kit (10X Genomics, 5’ Chromium) to facilitate single-cell RNA sequencing by Macrogen. After an initial cDNA quality assessment, the sequencing depth was determined based on the data quality and the number of cells captured. Subsequent sequencing was conducted to achieve greater depth following an initial evaluation of unique molecular identifiers (UMIs) and read counts. Sequencing data were processed using the 10X Genomics Cell Ranger 7.0.1 multi-pipeline, with reference transcriptomes GRCh38 (human) and mm10 (mouse) as the baseline. Data analysis was performed in R using the “Seurat” package. Cells that exhibited > 90% human gene content, < 20% mitochondrial gene content, and a minimum of 100 expressed genes were retained for analysis. Only genes that were represented in at least three cells were included. Cell doublets were identified and excluded using the “Scrublet” tool. For further analysis, the “presto,” “dittoSeq,” and “CellChat” packages in R were employed. Gene enrichment analysis was conducted using the ClueGO (Version 2.5.8) module within Cytoscape (Version 3.9.0) and the Gene Ontology (GO) immune system process database. Annotations inferred from electronic sources were excluded. The code supporting this study is publicly available at https://github.com/ytkim97/DA4505_Code/. The processed data is publicly available at https://figshare.com/s/539630820bc2e318ed6c. For scRNA-seq analysis, tumors were collected from independent biological samples across four treatment groups: vehicle (*n* = 2), pembrolizumab (*n* = 3), DA-4505 (*n* = 3), and combination treatment with pembrolizumab plus DA-4505 (COMBI; *n* = 4). Each sample represented an independent mouse-derived tumor specimen. Single cells were used for clustering, annotation, and cell-state characterization, whereas the independent tumor sample/mouse was considered the biological replicate for group-level interpretation.

### Immune cell isolation

Spleens were harvested from AhR KO mice, and their immune cells were isolated. Monocytes (#130-100-629, Miltenyi Biotec) was isolated using magnetic-activated cell sorting (MACS) with specific antibodies against surface markers. Briefly, spleen cells were first enriched using a MACS pan-T cell isolation kit, followed by the separation of monocytes with appropriate magnetic beads. The purity of the isolated cell populations was verified using flow cytometry. Bone marrow was collected from AhR KO mice and cultured in RPMI-1640 medium supplemented with 10% fetal bovine serum (FBS), 1% penicillin-streptomycin, and specific cytokines (GM-CSF for dendritic cells and M-CSF for macrophages) for dendritic cell (DC) and macrophage differentiation. Cells were cultured for 7 days, with media changes every 2–3 days, to allow differentiation into DCs and macrophages, respectively [32, 33].

### RNA extraction

Total RNA was extracted from cells using the RNeasy Mini Kit (Qiagen, Valencia, CA) according to the manufacturer’s instructions. The RNA concentration was measured using a NanoDrop 1000 spectrophotometer (ThermoFisher).

### RT-PCR & qPCR

cDNA was generated using the reverse transcriptase premix (#EBT-1515, ELPIS; KOR), and gene expression was quantified using real-time PCR with PowerUp™ SYBR™ Green Master Mix (#A25742, ThermoFisher). Gene-specific primers were as follows: For mouse, AhR (forward 5ʹ-CGCTGAAACATGAGCAAATTGG-3ʹ) and (reverse 5ʹ- ACAGCTTAGGTGCTGAGTCACAGG-3ʹ), and GAPDH (forward 5ʹ- AGGTCGGTGTGAACGGATTTG-3ʹ) and (reverse 5ʹ- TGTAGACCATGTAGTTGAGGTCA-3ʹ).

### Measurement of plasma cytokines by ELISA

At the experimental endpoint of the humanized mouse study, blood was collected into EDTA tubes (#365974, BD Biosciences), and plasma was obtained for cytokine analysis. Plasma levels of human IL-6 and CCL20 were measured using the Human IL-6 DuoSet ELISA (catalog no. DY206, R&D Systems) and Human CCL20/MIP-3α Quantikine ELISA Kit (catalog no. DM3A00, R&D Systems), respectively, according to the manufacturer’s instructions.

## Quantification and statistical analysis

### Cell viability

A549 and H2228 cells (3 × 10^3^) were seeded into each well and cultured in RPMI-1640 medium containing 10% FBS and 1% antibiotic–antimycotic solution. SB3181 and Bay2416964 were added to each well at various concentrations, and the cells were incubated at 37 °C in a humidified atmosphere with 5% CO_2_. After 24 h of treatment, cell viability was assessed by measuring luminescence using the CellTiter-Glo assay (Promega, Madison, WI, USA).

### Kinase assay

A biochemical assay for an AhR inhibitor was performed, and a kinase profile was established using the half-maximal inhibitory concentration (IC_50_) profiler assay (Millipore, UK) and the kinase profiler protocol (Millipore, UK). The IC_50_ was calculated using nonlinear regression analysis with GraphPad Prism 10.0 (GraphPad Software, CA, USA).

### Lung metastasis analysis

Metastatic lung tissue samples were fixed in 10% formalin overnight, dehydrated, and embedded in paraffin. Section  4-µm thick were cut, mounted onto slides, and stained with hematoxylin and eosin (H&E). The stained tissue sections were examined using a microscope. Imaging, data capture, processing, and analysis were performed using the LEICA Bond RX system (Leica Biosystems). The metastatic region was calculated using ImageJ software (NIH, Bethesda, MD, RRID: SCR_003070).

### Statistical analysis

The data are presented as the mean ± standard error of the mean (SEM). Statistical analyses were performed using Prism 10.0 (GraphPad Software Inc., USA) and R software. Flow cytometry and IHC data were analyzed using a t-test in GraphPad Prism. For all in vivo experiments, the biological replicate and statistical unit was defined as an individual mouse. For tumor growth analyses, tumor volume measurements were obtained longitudinally from each mouse and analyzed at the mouse level using two-way ANOVA followed by Tukey’s multiple comparisons test. For flow cytometry and immune profiling analyses, immune cell frequencies or absolute cell numbers were first calculated for each individual mouse, and group comparisons were performed using these mouse-level values. Individual cells acquired by flow cytometry were not treated as independent biological replicates. Data are presented as mean ± SEM unless otherwise indicated. Correlation analysis was conducted using Pearson’s correlation coefficient and the log-rank test for survival analyses. A p-value < 0.05 was considered statistically significant.

## Results

### AhR conditional knockout induces systemic immune activation in normal state mouse

**Fig. 1:**
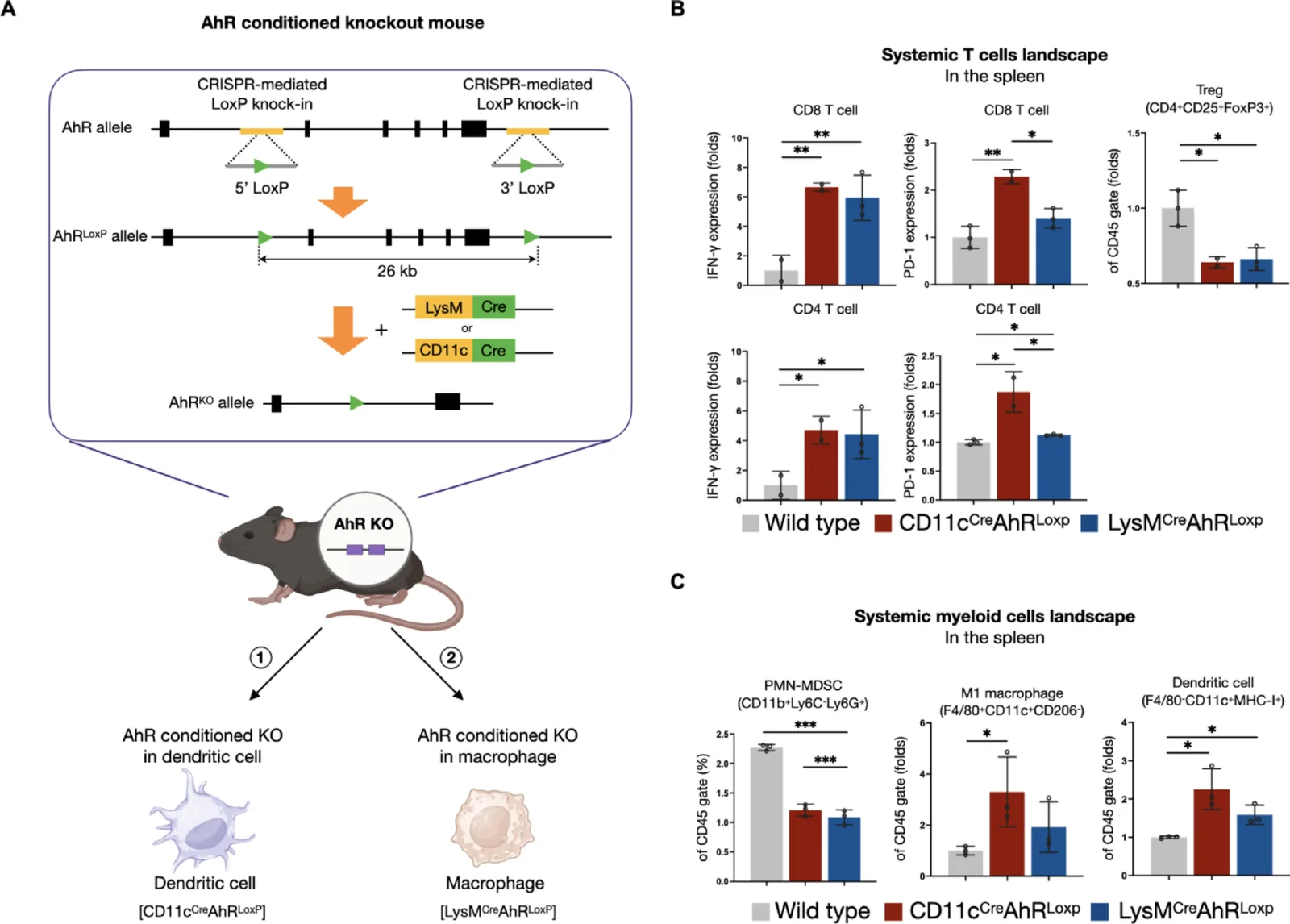
Conditional knockout of aryl hydrocarbon receptor in macrophage-lineage cells and dendritic cells promotes systemic immune activation under steady-state conditions. **A** Schematic design of AhR knockout mice using Cre-LoxP system in CD11c and Lysozyme, respectively. **B**, **C** Systemic immune cell status in the spleen. Flow cytometric analysis of splenic T-cell and myeloid populations in wild-type (*n* = 2–3), CD11c^Cre^-AhR^LoxP^ (*n* = 2–3), and LysM^Cre^-AhR^LoxP^ (*n* = 3) mice. Data represent mean ± SEM. Each data point represents one mouse, and statistical analyses were performed using mouse-level biological replicates. Individual cells acquired by flow cytometry were not treated as independent replicates. *P < 0.05, **P ≤ 0.01, ***P ≤ 0.001 by unpaired Student’s t-test

AhR plays a crucial role in the differentiation of CD8^+^ T cells [34–37]. Therefore, to determine whether AhR deletion in myeloid compartments alters systemic immune status, we generated two conditional AhR knockout mouse models using the Cre-LoxP system: CD11c^Cre^AhR^LoxP^ mice to target dendritic cell lineage (DCs) and LysM^Cre^AhR^LoxP^ mice to target macrophage lineage (MPs) cells (Figs. 1A and S1). To investigate the systemic immune modulation that occurred after AhR^KO^, spleens were harvested from wild-type (WT), CD11c^Cre^AhR^LoxP^ (AhR^KO^ in DCs), and LysM^Cre^AhR^LoxP^ (AhR^KO^ in MPs) mice and analyzed using flow cytometry. The AhR knockout (KO) in both DCs and MPs significantly enhanced IFN-γ expression in T cells and increased the abundance of dendritic cells compared with the WT group (Fig. 1B, C). The abundance of M1 macrophages and the PD-1 expressing T cells was significantly higher in the AhR^KO^ in DC group than in the WT group. A significant reduction in Treg and PMN-MDSC was observed in both AhR^KO^ groups compared with the WT group (Fig. 1B, C). These results demonstrate that conditional AhR deletion in dendritic cells or macrophage-lineage cells induces systemic immune activation under steady-state conditions, providing a baseline immunologic rationale for subsequent evaluation in tumor-bearing models.

### Conditional AhR ablation augments antitumor immunity via memory T-cell induction and alleviates immunosuppression

**Fig. 2:**
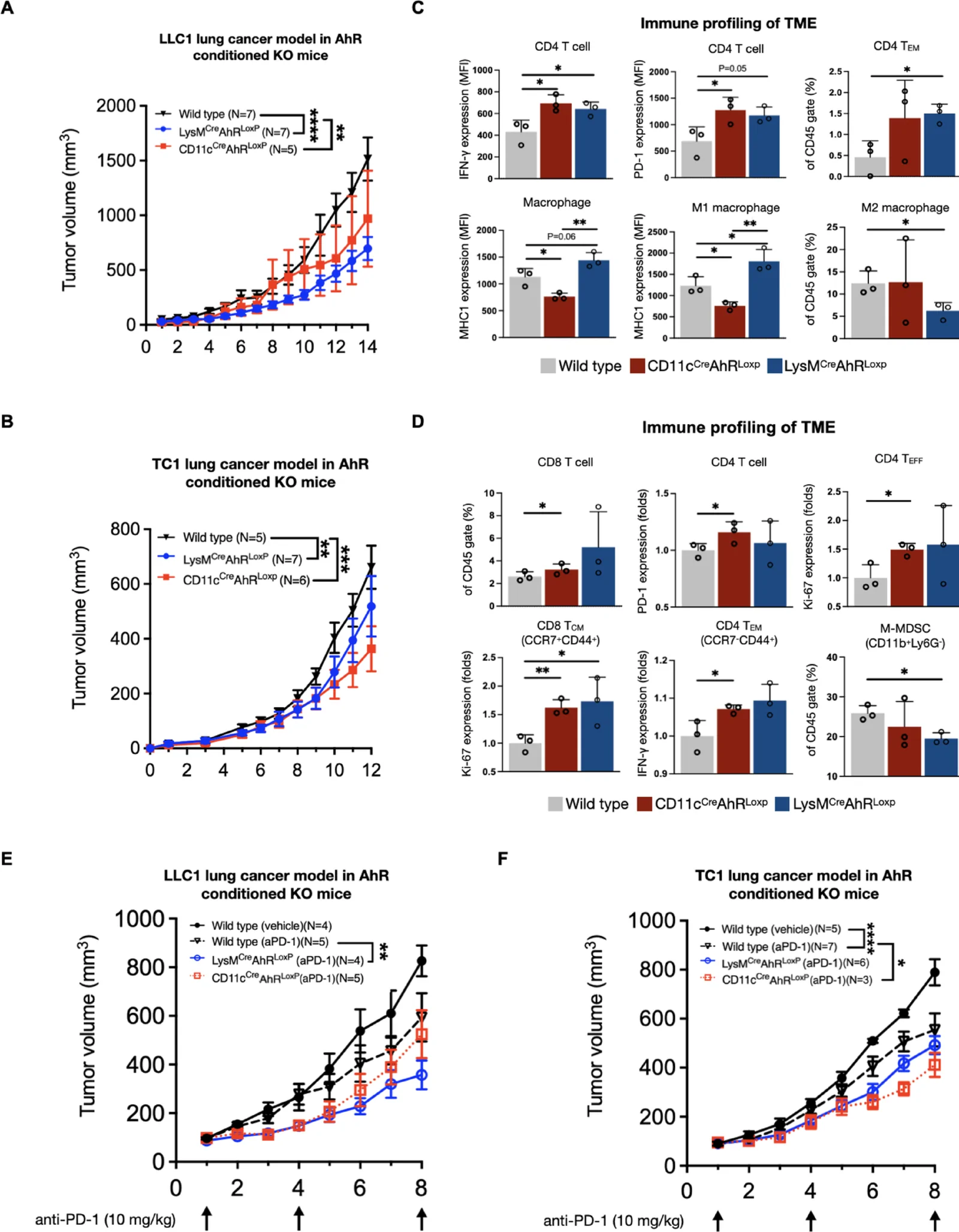
Macrophage and dendritic cell specific AhR deletion suppressed tumor growth and enhances the efficacy of PD-1 blockade. **A** Tumor growth in the LLC1 lung cancer model using wild-type (*n* = 7), LysM^Cre^-AhR^LoxP^ (*n* = 7) mice, and CD11c^Cre^-AhR^LoxP^ (*n* = 5). **B** Tumor growth in the TC1 lung cancer model using wild-type (*n* = 5), LysM^Cre^-AhR^LoxP^ (*n* = 7) mice, and CD11c^Cre^-AhR^LoxP^ (*n* = 6). **C** Flow cytometric analysis of tumor-infiltrating CD4⁺ T cells and macrophage populations in the LLC1 lung cancer model (*n* = 3 mice per group). **D** Flow cytometric analysis of tumor-infiltrating CD8⁺ T cells, CD4⁺ T-cell subsets, and M-MDSCs in the TC1 lung cancer model (*n* = 3 mice per group). MFI values are presented as fold change where indicated. **E** Tumor growth in the LLC1 lung cancer model following anti–PD-1 treatment (10 mg/kg) in wild-type vehicle-treated (*n* = 4), wild-type anti–PD-1-treated (*n* = 5), LysM^Cre^-AhR^LoxP^ anti–PD-1-treated (*n* = 4), and CD11c^Cre^-AhR^LoxP^ anti–PD-1-treated (*n* = 5) mice. **F** Tumor growth in the TC1 lung cancer model following anti–PD-1 treatment (10 mg/kg) in wild-type vehicle-treated (*n* = 5), wild-type anti–PD-1-treated (*n* = 7), LysM^Cre^-AhR^LoxP^ anti–PD-1-treated (*n* = 6), and CD11c^Cre^-AhR^LoxP^ anti–PD-1-treated (*n* = 3) mice. Arrows indicate the dosing schedule of anti–PD-1. Data are presented as mean ± SEM. Each data point represents one mouse, and statistical analyses were performed using mouse-level biological replicates. Individual cells acquired by flow cytometry were not treated as independent replicates. Statistical significance was determined by two-way ANOVA followed by Tukey’s multiple comparisons test for tumor growth analyses or by unpaired Student’s t-test for flow cytometry analyses. *P < 0.05, **P ≤ 0.01, ***P ≤ 0.001

To delineate the contribution of AhR to tumor immunity, we assessed tumor growth in WT, AhR^KO^ in DC, and AhR^KO^ in MP mice using both LLC1 and TC-1 lung cancer models. In the LLC1 model, tumor progression was significantly delayed in both conditional KO groups compared with that in the WT mice (Fig. 2A). A similar tumor suppression was confirmed in the TC-1 model (Fig. 2B). To investigate the immunological basis underlying these anti-tumor effects, tumor-infiltrating lymphocytes were analyzed using flow cytometry. Compared with those in the WT tumors, the AhR^KO^ in DCs and MPs exhibited increased effector-memory T cell infiltration and enhanced PD-1 and IFN-γ expression within CD4⁺ T cell subsets (Fig. 2C, D). Moreover, in the LLC1 model, AhR^KO^ in MPs resulted in a significant increase in MHC-I–expressing M1 macrophages and a reduction in M2 macrophages. In contrast, in the TC-1 model, proliferative CD8⁺ and CD4⁺ T cells were markedly enriched in the conditional KO groups. The frequency of monocytic MDSCs (M-MDSCs) was also significantly reduced in the conditional KO groups.

We evaluated the therapeutic implications of AhR deficiency in the context of immune checkpoint blockade. The LLC1 or TC-1 tumor bearing WT or conditional KO mice were treated with anti–PD-1 antibody. Tumor growth suppression was further enhanced in mice with AhR^KO^ in MPs and DCs than in WT, indicating a synergistic effect between AhR loss and PD-1 blockade (Fig. 2E, F). Notably, the AhR^KO^ in MP group effectively inhibited tumor growth in the LLC1 model, whereas the AhR^KO^ in DC group suppressed tumor growth in the TC-1 model (Fig. 2F). Collectively, these findings demonstrate that macrophage and dendritic cell specific deletion of AhR not only restrains tumor growth but also potentiates the efficacy of PD-1 immunotherapy by reshaping the tumor immune microenvironment toward enhanced T-cell infiltration and activation.

### AhR inhibition enhances antitumor immunity by boosting macrophage antigen presentation in ICI-refractory LLC1 model

**Fig. 3:**
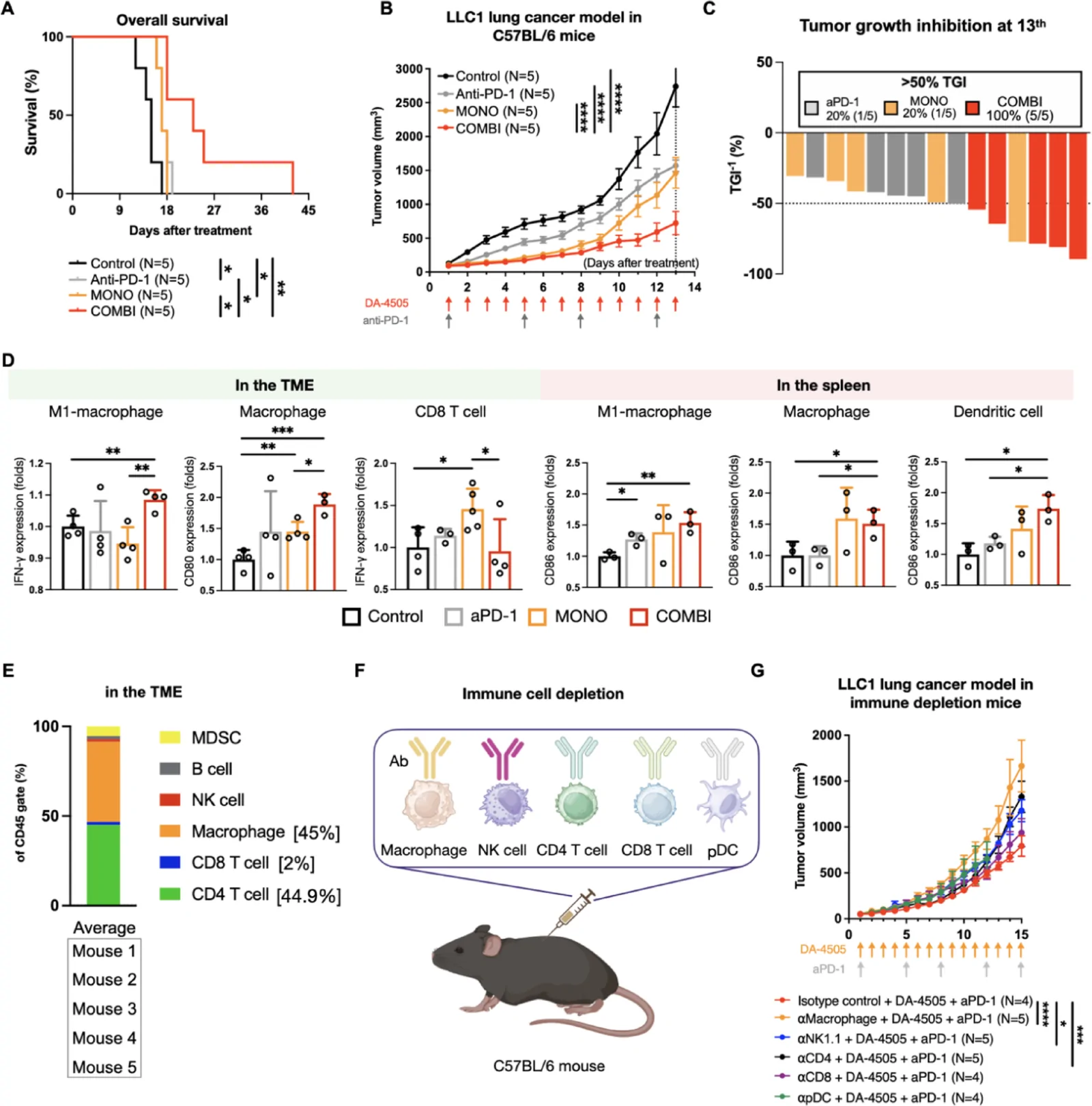
AhR blockade augments innate immunity in an ICI-refractory LLC1 model. **A** Overall survival of LLC1 tumor-bearing mice in the control, anti–PD-1, MONO, and COMBI groups (*n* = 5 mice per group). **B** Tumor growth in the LLC1 lung cancer model in C57BL/6 mice treated with control, anti–PD-1, MONO, or COMBI (*n* = 5 mice per group). **C** Tumor growth inhibition (TGI) at day 13 in the anti–PD-1, MONO, and COMBI groups (*n* = 5 mice per group). **D** Flow cytometric analysis of tumor-infiltrating and splenic immune cell populations following control, anti–PD-1, MONO, or COMBI treatment. Activation-associated markers were assessed in macrophage and T-cell populations in the tumor microenvironment and spleen. **E** Composition of tumor-infiltrating immune cell populations in the LLC1 tumor microenvironment (*n* = 5 mice). **F** Schematic representation of the immune cell depletion experiment targeting macrophages, NK cells, CD4⁺ T cells, CD8⁺ T cells, or pDCs in C57BL/6 mice. **G** Tumor growth in the LLC1 lung cancer model following immune cell depletion in mice treated with DA-4505 and anti–PD-1. Groups included isotype control (*n* = 4), macrophage-depleted (*n* = 5), NK cell-depleted (*n* = 5), CD4⁺ T-cell-depleted (*n* = 5), CD8⁺ T-cell-depleted (*n* = 4), and pDC-depleted (*n* = 4) mice. Arrows indicate the dosing schedule of anti–PD-1 or DA-4505. Data are presented as mean ± SEM. Each data point represents one mouse, and statistical analyses were performed using mouse-level biological replicates. Individual cells acquired by flow cytometry were not treated as independent replicates. Statistical significance was determined by two-way ANOVA followed by Tukey’s multiple comparisons test for tumor growth analyses or by unpaired Student’s t-test for flow cytometry analyses. *P < 0.05, **P ≤ 0.01, ***P ≤ 0.001

To evaluate the reproducibility of the observed anti-tumor effects, we established a C57BL/6 mouse model inoculated with the ICI-refractory LLC1 lung cancer cell line. AhR inhibition was achieved using DA-4505, a novel pharmacological AhR inhibitor selected for in vivo therapeutic assessment based on the potency, selectivity, and minimal direct cytotoxicity of its active form, SB3181 (Fig. S3). The mice were treated with DA-4505 monotherapy (MONO) and the DA-4505 plus anti-PD-1 (COMBI) regimen. The COMBI group exhibited a significant improvement in survival and enhanced anti-tumor effects compared with the other groups (Fig. 3A–C). Notably, these effects were exclusive to the COMBI group. To explore the underlying mechanisms of these differences, we performed immune cell profiling in both the TME and spleen. While DCs and MPs in the spleen were systemically activated by AhR inhibition, a significant increase in MP activation within the TME was observed in the COMBI group (Fig. 3D). This indicates that MPs in the TME have crucial anti-tumor effects.

To further investigate the immune components in the TME of LLC1, we obtained untreated LLC1 tumors and conducted a comprehensive analysis of the major immune cell populations. Among the total immune cells, MPs accounted for 45%, whereas CD8 T cells comprised 2% (Fig. 3E). Based on these results, we hypothesized that the anti-tumor effects observed in Fig. 2E are MP-mediated. To test the relative contribution of immune-cell subsets, we performed immune cell depletion assays. In the LLC1 model, MP depletion produced the most pronounced attenuation of tumor control, whereas NK-cell and CD4⁺ T cell depletion also increased tumor growth to a lesser extent, and CD8⁺ T cell depletion had a comparatively smaller effect (Fig. 3F, G). These findings suggest that MP remodeling is a major contributor to the antitumor effects of AhR inhibition in the ICI-refractory LLC1 model, while also supporting the involvement of additional immune subsets, including NK cells and CD4⁺ T cells.

### AhR blockade enhances CD8⁺ T cell–mediated immunity and potentiates immuno- and chemotherapy in an ICI-sensitive TC-1 model

**Fig. 4:**
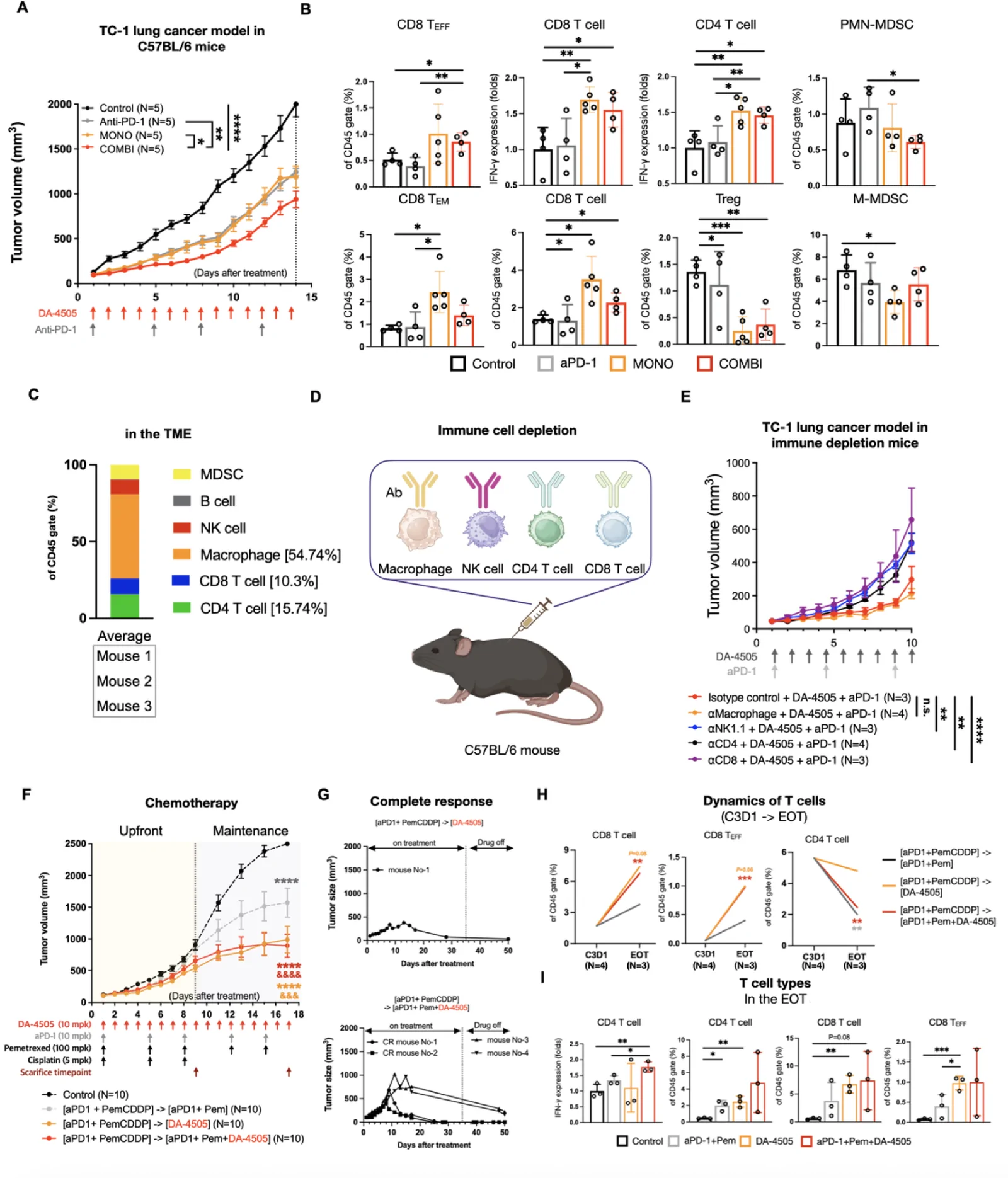
AhR blockade enhances adaptive immunity in an ICI-sensitive TC-1 model. **A** Tumor growth in the TC-1 tumor model in C57BL/6 mice treated with control, anti–PD-1, MONO, or COMBI (*n* = 5 mice per group). **B** Flow cytometric analysis of tumor-infiltrating immune cell populations following control, anti–PD-1, MONO, or COMBI treatment. CD8⁺ and CD4⁺ T-cell subsets and MDSC populations were analyzed. **C** Composition of tumor-infiltrating immune cell populations in the TC-1 tumor microenvironment (*n* = 3 mice). **D** Schematic representation of the immune cell depletion experiment targeting macrophages, NK cells, CD4⁺ T cells, or CD8⁺ T cells in C57BL/6 mice. **E** Tumor growth in the TC-1 tumor model following immune cell depletion in mice treated with DA-4505 and anti–PD-1. Groups included isotype control (*n* = 3), macrophage-depleted (*n* = 4), NK cell-depleted (*n* = 3), CD4⁺ T-cell-depleted (*n* = 4), and CD8⁺ T-cell-depleted (*n* = 3) mice. **F** Tumor growth during upfront and maintenance treatment in the TC-1 tumor model. Mice received anti–PD-1, pemetrexed, and cisplatin as upfront treatment, followed by maintenance treatment with anti–PD-1 plus pemetrexed, DA-4505, or anti–PD-1 plus pemetrexed and DA-4505 (*n* = 10 mice per group). An untreated control group was included (*n* = 10). **G** Individual tumor growth curves of mice from the DA-4505-containing maintenance treatment groups during treatment and following drug withdrawal, showing mice that achieved complete responses. **H** Changes in CD8⁺ T cells, CD8⁺ T_EFF_ cells, and CD4⁺ T cells between C3D1 (*n* = 4) and end of treatment (EOT; *n* = 3) during maintenance therapy. **I** Flow cytometric analysis of T-cell populations at EOT following control, anti–PD-1 plus pemetrexed, DA-4505, or anti–PD-1 plus pemetrexed and DA-4505 maintenance treatment (*n* = 3 mice per group). CD4⁺ T-cell IFN-γ expression, CD4⁺ and CD8⁺ T-cell proportions, and CD8⁺ T_EFF_ populations were assessed. Arrows indicate the dosing schedule of anti–PD-1 or DA-4505. Data are presented as mean ± SEM. Each data point represents one mouse, and statistical analyses were performed using mouse-level biological replicates. Individual cells acquired by flow cytometry were not treated as independent replicates. Statistical significance was determined by two-way ANOVA followed by Tukey’s multiple comparisons test for tumor growth analyses or by unpaired Student’s t-test for flow cytometry analyses. *P < 0.05, **P ≤ 0.01, ***P ≤ 0.001

To determine if combining ICI with an AhR inhibitor could induce stronger anti-tumor effects in an ICI-sensitive model, we verified the anti-tumor effects in the ICI-responsive TC-1 model. Compared with the control group, significant tumor size reduction was observed in the aPD-1, MONO, and COMBI groups, with the COMBI group demonstrating the best anti-tumor effects compared with the aPD-1 and MONO groups (Fig. 4A and S4A–S4C). After confirming that the anti-tumor effects were immune response-driven, we performed immune cell profiling within the TME (Fig. 4B). The increased abundance of CD8 + T cells and their enhanced differentiation toward effector cells (T_EFF_) together with decreased Tregs and MDSCs, suggest that AhR inhibition relieves immune suppression and synergizes with ICI-driven adaptive immunity to produce potent antitumor effects (Fig. 4B). Specifically, the TME of TC-1 exhibited a five-fold increase in the proportion of CD8⁺ T cells compared with that of LLC1 (Fig. 4C). Immune-cell depletion assays were conducted to explore these differences. The results showed that CD8⁺ T cell depletion produced the most pronounced reversal of tumor control in the TC-1 model, whereas CD4⁺ T cell and NK cell depletion also increased tumor growth, and MP depletion had a comparatively smaller effect (Fig. 4D, E).

However, despite being an ICI-sensitive model, no complete response was observed in any treatment group. Given this partial efficacy and considering that chemotherapy can induce immunogenic cell death (ICD) and promote immune priming within the tumor microenvironment, we evaluated combination treatment with chemotherapy (Figs. 4 F and S4D–S4E). Setting cisplatin plus pemetrexed plus aPD-1 as the standard of care (SoC), we administered either DA-4505 as an add-on or DA-4505 alone during the maintenance phase. Both treatments resulted in significant anti-tumor effects compared with the [SoC -> SoC] regimen. Notably, complete responses were observed in both the DA-4505 alone (1/9) and DA-4505 add-on (2/10) groups, and these responses were sustained even after treatment cessation (Fig. 4G). The DA-4505 add-on group, a subset of mice (2/10), exhibited continued tumor regression even after treatment. To assess whether the enhanced anti-tumor response resulted from chemotherapy-induced immune activation, we performed immune profiling of tumor tissues collected at interim and end points (EOT). Following SoC treatment at an interim point (C3D1), CD80 expression on DCs and M1 macrophages significantly increased, accompanied by an expansion of DCs (Fig. S4F). During maintenance therapy, treatment with DA-4505 alone or in combination further elevated the CD8^+^ T cell and CD8^+^ T_EFF_ abundance at EOT relative to C3D1 (Fig. 4H). Compared with the SoC groups, the DA-4505 and SoC plus DA-4505 groups showed marked enrichment of CD4⁺ and CD8⁺ T_EFF_. Furthermore, IFN-γ expression in CD4⁺ T cells were significantly higher in the SoC plus DA-4505 group (Fig. 4I). These findings suggest that AhR inhibition-driven T-cell differentiation and activation underlie the anti-tumor effects observed in the TC-1 model. Enhanced antigen presentation induced by chemotherapy further amplifies this response, providing a strong immune boost that synergizes with AhR inhibition to achieve complete tumor regression.

### AhR inhibition shows broader therapeutic activity across colorectal cancer and metastatic TNBC models

To further examine whether the therapeutic effects of AhR inhibition extend across additional syngeneic tumor contexts, we evaluated the CT26 colorectal cancer model and the 4T1 orthotopic TNBC model [38]. In the CT26 model, COMBI treatment showed the strongest tumor growth inhibition, durable complete responses after treatment withdrawal, and a significant survival benefit (Fig. S5A–S5C). Immune profiling revealed increased T-cell abundance, enhanced CD8⁺ effector-memory T-cell differentiation, a similar trend in CD4⁺ T cells, and increased activation of DCs and macrophages, particularly in the COMBI group (Fig. S5D–S5E). Immune-cell depletion further supported a major contribution of CD8⁺ T cells to tumor control, whereas macrophage depletion resulted in a milder increase in tumor growth (Fig. S5F–S5H).

We next evaluated AhR inhibition in the 4T1 orthotopic TNBC model, which is characterized by aggressive tumor growth and spontaneous lung metastasis. In this model, COMBI treatment enhanced primary tumor growth inhibition, improved survival, and reduced spontaneous lung metastatic burden (Fig. S6A–S6E). In a perioperative surgery setting, COMBI treatment reduced tumor relapse and improved overall survival after tumor resection, accompanied by increased infiltration of CD4⁺ T cells and macrophages in tumor tissues collected at surgery (Fig. S6F–S6J). Together, these findings support the broader therapeutic activity of AhR inhibition across colorectal cancer and metastatic TNBC settings, with context-dependent contributions from T-cell activation and myeloid-cell remodeling.

### AhR blockade supports CD8⁺ TEMRA expansion and reduced Treg enrichment in an ICI-refractory humanized PDX model

**Fig. 5:**
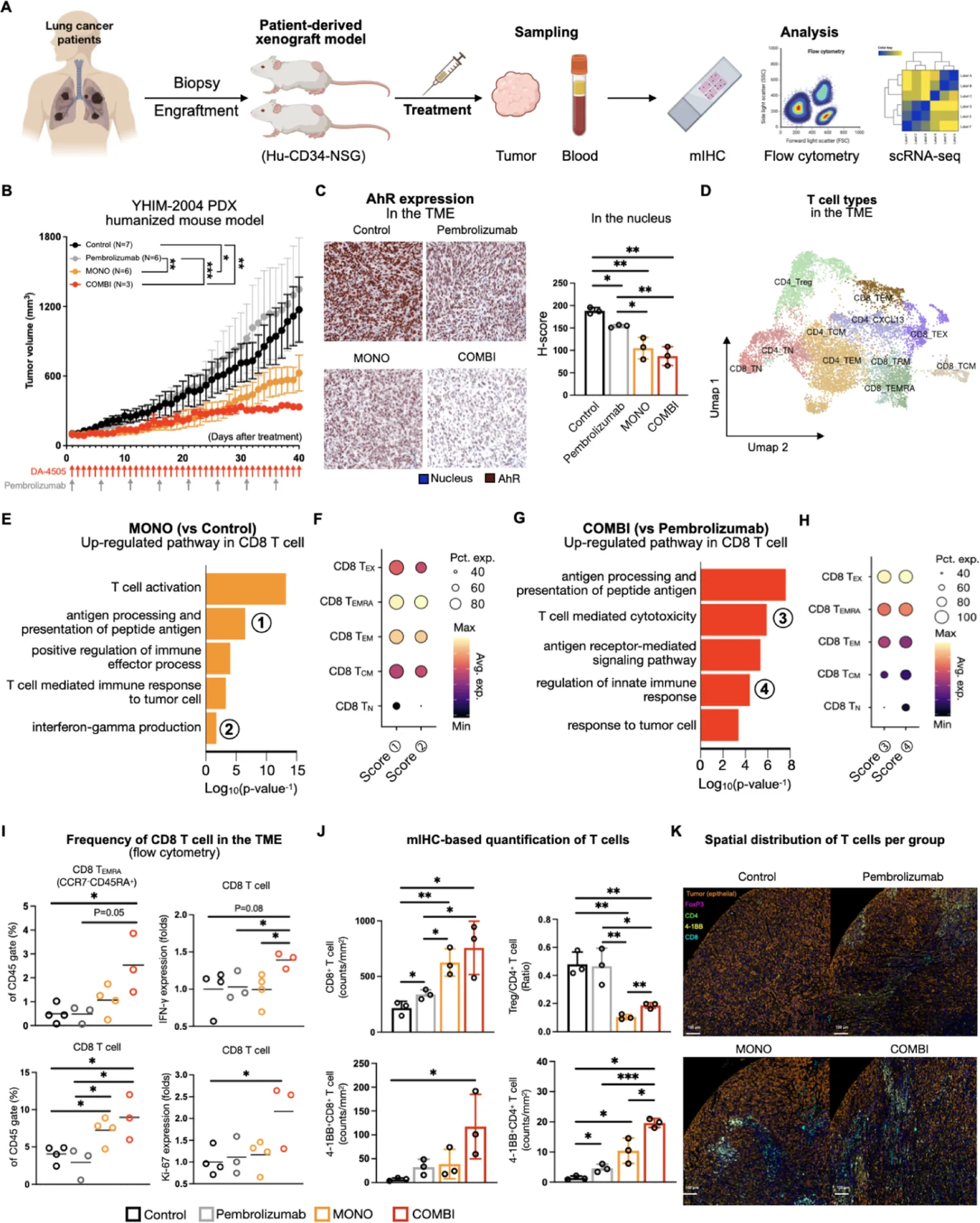
Humanized PDX analysis supports AhR inhibition-associated T-cell remodeling in an ICI-refractory tumor context. **A** Schematic overview of the study design and experimental workflow. Lung cancer patient-derived tumors were engrafted into humanized Hu-CD34-NSG mice, followed by treatment, tumor and blood sampling, and analysis by multiplex immunohistochemistry (mIHC), flow cytometry, and single-cell RNA sequencing (scRNA-seq). **B** Tumor growth in the YHIM-2004 patient-derived xenograft (PDX) humanized mouse model treated with control (*n* = 7), pembrolizumab (*n* = 6), MONO (*n* = 6), or COMBI (*n* = 3). **C** Representative immunohistochemical staining of AhR in tumor tissues from the control, pembrolizumab, MONO, and COMBI groups (*n* = 3 mice per group). Brown staining indicates AhR expression, and blue staining indicates hematoxylin-counterstained nuclei. Nuclear AhR expression was quantified using the H-score. **D** UMAP visualization of tumor-infiltrating T cells identified by scRNA-seq, showing 11 T-cell subclusters. **E** Pathway enrichment analysis of genes upregulated in CD8⁺ T cells from the MONO group compared with the control group. Differentially expressed genes were defined by an average log2 fold-change > 0.1 and *P* < 0.05. **F** Gene set scores for the MONO-associated pathways identified in (E) across CD8⁺ T-cell subtypes. **G** Pathway enrichment analysis of genes upregulated in CD8⁺ T cells from the COMBI group compared with the pembrolizumab group. Differentially expressed genes were defined by an average log2 fold-change > 0.2 and *P* < 0.05. **H** Gene set scores for the COMBI-associated pathways identified in (G) across CD8⁺ T-cell subtypes. **I** Flow cytometric analysis of tumor-infiltrating CD8⁺ T cells in the control (*n* = 4), pembrolizumab (*n* = 3), MONO (*n* = 4), and COMBI (*n* = 3) groups. The frequency of CD8⁺ T cells and CCR7⁻CD45RA⁺ CD8 T_EMRA_ cells, together with IFN-γ and Ki-67 expression in CD8⁺ T cells, was assessed. **J** mIHC-based quantification of tumor-infiltrating T-cell populations in the control, pembrolizumab, MONO, and COMBI groups (*n* = 3 mice per group). CD8⁺ T-cell density, Treg/CD4⁺ T-cell ratio, 4-1BB⁺CD8⁺ T-cell density, and 4-1BB⁺CD4⁺ T-cell density were quantified. **K** Representative mIHC images showing the spatial distribution of T-cell populations in tumor tissues from the control, pembrolizumab, MONO, and COMBI groups. Tumor epithelial cells, FoxP3, CD4, 4-1BB, and CD8 are shown by the indicated markers. Arrows indicate the dosing schedule of pembrolizumab or DA-4505. Data are presented as mean ± SEM. Each data point represents one mouse, and statistical analyses were performed using mouse-level biological replicates. Individual cells acquired by flow cytometry and IHC were not treated as independent replicates. Statistical significance was determined by two-way ANOVA followed by Tukey’s multiple comparisons test for tumor growth analyses or by unpaired Student’s t-test for flow cytometry and H-score analyses. *P < 0.05, **P ≤ 0.01, ***P ≤ 0.001.; LUSC, lung squamous cell carcinoma; ANOVA, analysis of variance; Green color, CD8 T cell; Blue color, DAPI; White color, Cancer cell

We hypothesized that AhR inhibition would enhance immune cell activation within the TME. To investigate immune-cell dynamic variation, we used the PDX model, YHIM-2004 (lung squamous cell carcinoma: LUSC), engrafted in humanized mice. The analysis followed the experimental workflow presented in Fig. 5A. Despite the lack of response to immune checkpoint inhibitor (ICI) treatment, both MONO and COMBI groups demonstrated significant tumor reduction compared with the control and pembrolizumab groups (Figs. 5B and S7A). These findings were accompanied by immune-cell changes consistent with AhR inhibition-associated immune remodeling (Figs. 5C and S7B–S7C). To identify the specific immune-cells involved, we interrogated immune-cell states (activation, exhaustion, memory) and their quantitative shifts. First, we conducted a detailed exploration of T cell compartment within the TME using single-cell RNA sequencing (scRNAseq) analysis. By sub-clustering 3,767 intra-tumoral T cells, we identified 11 T cell phenoptypes (Figs. 5D and S7D). Six unique clusters (CD8 T_N_, CD8 T_CM_, CD8 T_EM_, CD8 T_RM_, CD8 T_EX_, CD8 T_EMRA_) were also identified using canonical markers (Table S1). Differential gene-expression analysis followed by pathway enrichment was conducted to define the features of CD8⁺ T cells enhanced by AhR inhibition. Relative to the control, the MONO group showed significant pathway enrichment linked to T-cell activation, tumor-cell killing, and memory-like differentiation, including antigen processing, peptide antigen presentation, and IFN-γ production (Fig. 5E–F). The COMBI versus pembrolizumab contrast showed similar pathway patterns, consistent with AhR inhibition-associated T-cell remodeling. In particular, the upregulation of T cell–mediated cytotoxicity and innate immune response pathways supports a model in which interactions with myeloid cells support sustained CD8⁺ T-cell functionality (Fig. 5G–H).

Notably, in vitro flow cytometry analysis for T cell profiling on the same tumor tissue revealed that AhR inhibition significantly increased the abundance of CD8^+^ T_EMRA_ and CD8^+^ T cells (Figs. 5I and S7E). Furthermore, substantial increases in CD8^+^ T cell proliferation (Ki-67 expression) and activation (IFN-γ expression) provided additional evidence supporting the scRNAseq results (Fig. 5I). Consistent with the transcriptional and cytometric data, in situ spatial profiling by multiplex IHC (mIHC) showed a reshaped T-cell compartment under AhR blockade. Compared with the control and pembrolizumab, both the MONO and COMBI groups exhibited higher intratumoral CD8⁺ T-cell density, increased frequencies of 4-1BB⁺ (CD137⁺) CD8⁺ and CD4⁺ T cells, and a lower Treg/CD4⁺ ratio, with spatial maps revealing deeper CD8⁺ infiltration into tumor nests (Fig. 5J–K). Overall, these data provide supportive evidence that AhR inhibition is associated with enhanced T-cell differentiation and activation, with a context-dependent contribution from myeloid/APC remodeling that may support T-cell functionality in selected tumor microenvironments.

### AhR inhibition reprograms macrophages to relieve Treg-mediated suppression

**Fig. 6:**
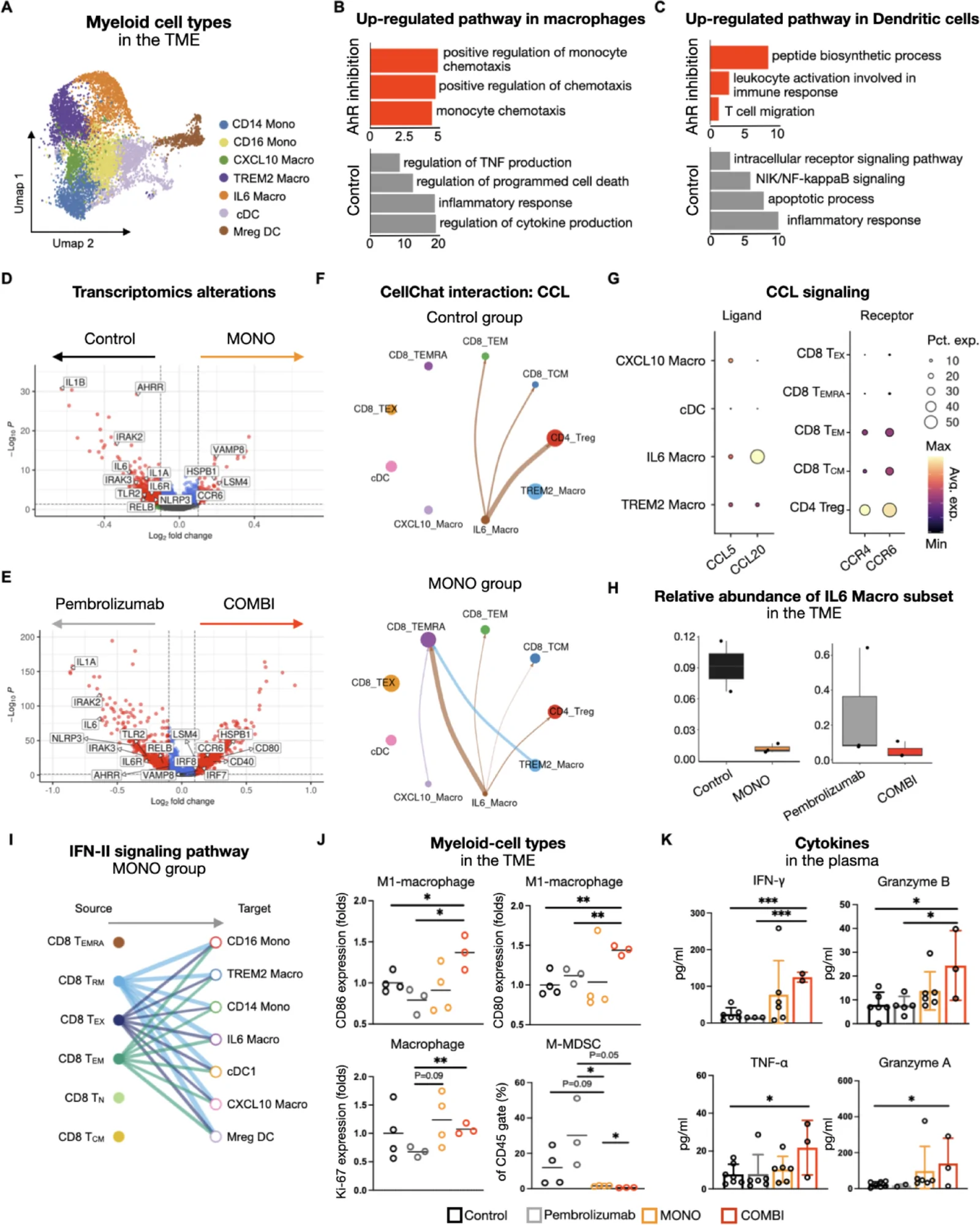
AhR inhibition reshapes myeloid subclusters and modulates myeloid–T cell crosstalk, synergizing with anti-PD-1 therapy. **A** UMAP visualization of tumor-infiltrating myeloid cells identified by scRNA-seq, showing seven myeloid cell subclusters. **B**, **C** Pathway enrichment analysis of differentially expressed genes in macrophages (**B**) and dendritic cells (**C**) comparing AhR inhibition with the control condition. Differentially expressed genes used for pathway enrichment analysis were defined by an average log2 fold-change > 0.15 and *P* < 0.05. **D**, **E** Volcano plots showing differential gene expression in tumor-infiltrating myeloid cells between the MONO and control groups (**D**) and between the COMBI and pembrolizumab groups (**E**). **F** CellChat-inferred CCL signaling networks among tumor-infiltrating immune cell populations in the control and MONO groups. **G** Dot plot showing the expression of selected CCL ligands in myeloid cell subsets and their corresponding receptors in T-cell subsets. **H** Relative abundance of the IL6 macrophage subset in the tumor microenvironment in the control versus MONO and pembrolizumab versus COMBI groups. **I** CellChat-inferred IFN-II signaling network between T-cell and myeloid cell subsets in the MONO group. **J** Flow cytometric analysis of tumor-infiltrating myeloid cell populations in the control (*n* = 4), pembrolizumab (*n* = 3), MONO (*n* = 4), and COMBI (*n* = 3) groups. CD86 and CD80 expression in M1 macrophages, Ki-67 expression in macrophages, and the frequency of M-MDSCs were assessed. **K** Plasma concentrations of IFN-γ, TNF-α, granzyme A, and granzyme B in the control (*n* = 6–7), pembrolizumab (*n* = 2–6), MONO (*n* = 5–6), and COMBI (*n* = 2–3) groups. Statistical significance was determined by two-way ANOVA followed by Tukey’s multiple comparisons test for tumor growth analyses or by unpaired Student’s t-test for flow cytometry analyses. *P < 0.05, **P ≤ 0.01, ***P ≤ 0.001.; CD14 Mono, CD14^+^ monocyte; CD16 Mono, CD16^+^ monocyte; TREM2 Macro, TREM2^+^ macrophage; IL6 Macro, IL6^+^ macrophage; CXCL10 Macro, CXCL10^+^ macrophage

To investigate the impact of AhR inhibition on myeloid cells within the TME, we performed sub-clustering of 8,446 intratumoral myeloid cells, identifying 7 distinct subsets (Figs. 6A and S8, Table S2). In macrophages, AhR inhibition downregulated inflammatory pathways while upregulating chemotaxis-related genes (Fig. 6B). Similarly, dendritic cells showed enhanced immune activation and T-cell migration programs with reduced inflammatory signaling (Fig. 6C). In both macrophages and dendritic cells, pathways associated with cell death were downregulated. Consistently, both the monotherapy and combination groups exhibited decreased expression of AhR target genes (*AHRR*) and pro-inflammatory cytokines (*IL1A*,* IL1B*,* IL6*), but increased chemotactic and activation markers such as *CD40* and *CD80* (Fig. 6D–E). Cell–cell interaction analysis revealed that IL6⁺ macrophages engaged CD4⁺ Tregs via *CCL* ligands, and this interaction was markedly diminished upon AhR inhibition (Fig. 6F–G). Consistent with these findings, ELISA analysis of plasma from humanized PDX-bearing mice demonstrated significantly reduced levels of CCL20 and IL-6 following pharmacologic AhR inhibition with DA-4505 (Fig. S9).

Notably, the frequency of IL6-macrophages was also reduced, indicating that suppression of this subset limits Treg recruitment (Fig. 6H). Moreover, type II interferon signaling between CD8⁺ T cells and myeloid subsets was selectively enriched in the MONO group (Fig. 6I), suggesting enhanced CD8⁺ T-cell activation secondary to Treg reduction. Flow cytometry analysis confirmed increased M1-macrophage activation (CD80, CD86), elevated macrophage proliferation, and reduced M-MDSCs (Fig. 6J). Systemically, plasma levels of IFN-γ, TNF-α, and granzymes were elevated, indicating enhanced effector responses (Fig. 6K). Taken together, these findings suggest that AhR inhibition is associated with reduced IL6⁺ macrophage abundance, decreased circulating IL-6 and CCL20 levels, attenuated Treg-associated signaling, and enhanced chemotaxis and activation programs, consistent with increased CD8⁺ T-cell effector function and antitumor immunity.

### Translational validation of AhR inhibition–driven immune remodeling and its prognostic relevance in human cancer cohorts

**Fig. 7:**
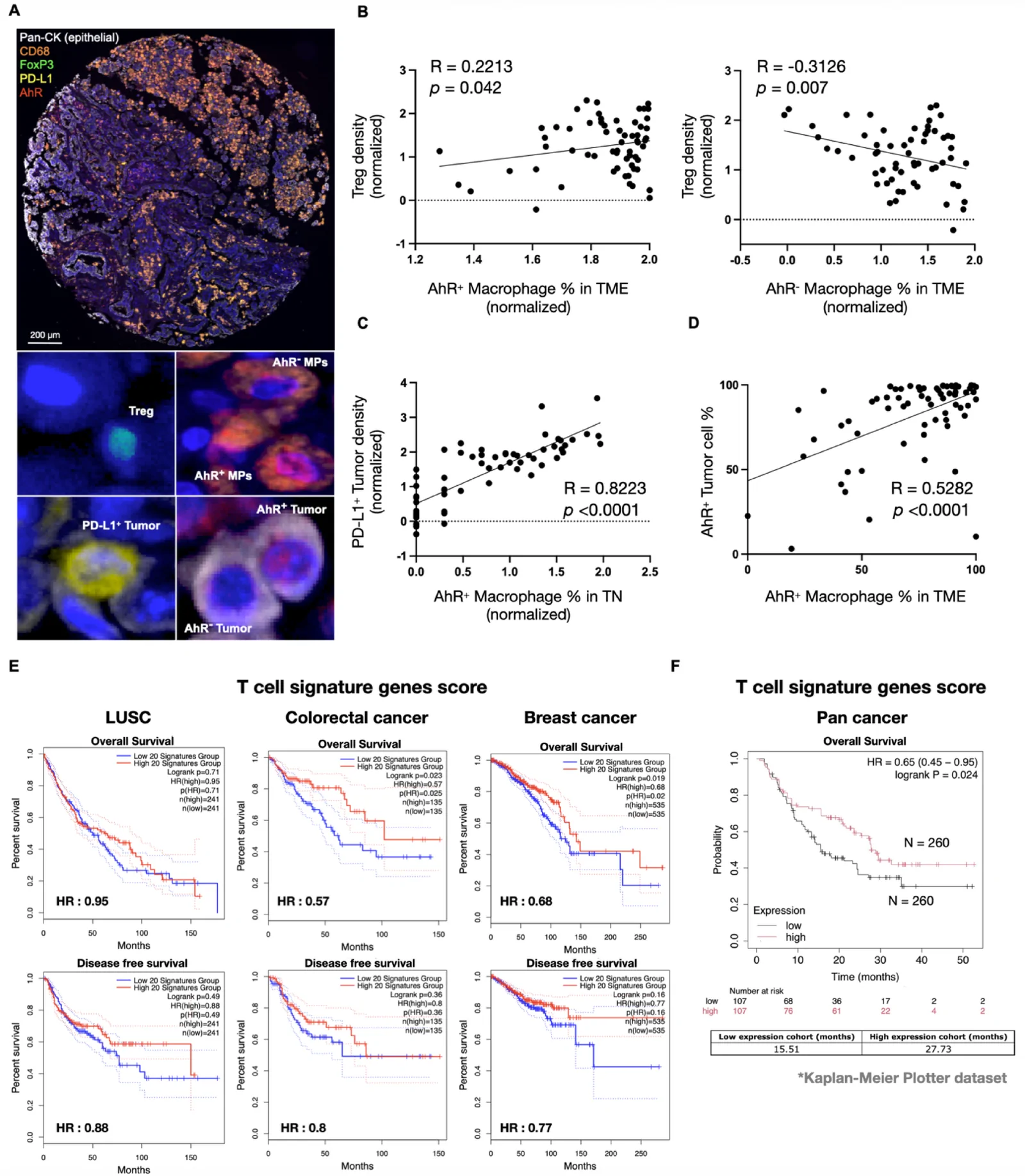
Translational relevance of AhR-dependent immune modulation in human NSCLC and validation of AhR inhibition–associated T-cell signatures in TCGA survival datasets. **A** Representative multiplex immunohistochemistry (mIHC) images of human NSCLC tissue microarrays stained for Pan-CK (white), CD68 (orange), FOXP3 (green), PD-L1 (yellow), AhR (red), and DAPI (blue). Representative magnified images show Tregs, AhR⁺ macrophages, PD-L1⁺ tumor cells, and AhR⁺ and AhR⁻ tumor cells. Scale bar, 200 μm. **B** Correlation analyses between the proportions of AhR⁺ or AhR⁻ macrophages in the tumor microenvironment (TME) and normalized Treg density. **C** Correlation analysis between the proportion of AhR⁺ macrophages in the tumor nest (TN) and normalized PD-L1⁺ tumor cell density. **D** Correlation analysis between the proportion of AhR⁺ macrophage d des in the TME and the proportion of AhR⁺ tumor cells. **E** Validation of the AhR inhibition-associated T-cell signature gene set derived from scRNA-seq analysis in Fig. 5 using TCGA patient cohorts. Kaplan–Meier analyses of overall survival and disease-free survival according to T-cell signature gene scores in LUSC, colorectal cancer, and breast cancer cohorts. **F** Kaplan–Meier analysis of overall survival according to the AhR inhibition-associated T-cell signature gene score in a pan-cancer cohort of patients treated with anti–PD-1 therapy. Patients were stratified into high- and low-signature groups. Hazard ratio (HR), log-rank P value, and sample numbers are indicated in the plot. Data were obtained from the Kaplan–Meier Plotter dataset. For (**B**–**D**), correlations were assessed using Pearson’s correlation analysis. For (**E**, **F**), survival differences between groups were evaluated using the log-rank test

To evaluate the clinical relevance of AhR inhibition-driven immune modulation observed in our preclinical models, we analyzed a human non-small cell lung cancer tissue microarray (TMA) using mIHC for AhR, CD68, FOXP3, PD-L1, and Pan-CK (Fig. 7A, Table S3). Quantitative analysis revealed a significant positive correlation between the proportion of AhR⁺ macrophages and the density of FOXP3⁺ Tregs within the TME. In contrast, AhR⁻ macrophages showed a negative association with Treg abundance (Fig. 7B). Furthermore, AhR⁺ macrophages in the tumor nest correlated strongly with PD-L1⁺ tumor cell density. In contrast, their abundance in the TME was positively associated with the proportion of AhR⁺ tumor cells (Fig. 7C–D). These findings suggest that AhR⁺ macrophage enrichment is associated with immunosuppressive features of the human NSCLC TME, including PD-L1 expression and regulatory T-cell enrichment.

To extend these findings, we investigated whether the transcriptional program induced by AhR inhibition in humanized scRNA-seq datasets was also relevant to clinical outcomes. We identified a set of AhR inhibition–responsive T-cell signature genes that were associated with enhanced effector and memory differentiation (Table S4). Survival analyses using TCGA cohorts revealed that higher expression of this T-cell signature was significantly associated with improved overall and disease-free survival in colorectal and breast cancers, with a similar trend observed in LUSC datasets (Fig. 7E). However, unlike the T-cell signature, expression of the myeloid signature did not show a significant association with patient survival across cancer types, although a modest trend toward improved survival was observed (Fig. S10). Notably, in patients treated with anti–PD-1 therapy, high expression of the AhR inhibition–induced T-cell signature was associated with significantly prolonged overall survival (HR = 0.65, *p* = 0.024; Fig. 7F, Table S5).

Collectively, these correlative analyses suggest an association between AhR inhibition–associated T-cell transcriptional programs and favorable clinical outcomes in human tumors. While these findings do not establish causality, they are consistent with the immune-modulatory effects observed in murine and humanized models and support further investigation of AhR blockade as a potential strategy to enhance immune checkpoint therapy.

## Discussion

In this study, we investigated whether AhR inhibition can reprogram antitumor immunity and enhance therapeutic responses across distinct tumor models [39–41]. Although AhR has been implicated in tumor immune suppression, the cellular mechanisms by which AhR blockade reshapes systemic and local immunity, particularly in immune checkpoint inhibitor-resistant tumors, remain incompletely defined. Our findings demonstrate that AhR inhibition converts the tumor microenvironment from an immunosuppressive to an immune-stimulatory state by coordinating myeloid-cell remodeling and T-cell activation.

Using LysM-Cre and CD11c-Cre drivers, which have been widely used to induce conditional gene deletion in macrophage-lineage and dendritic-cell-enriched compartments, respectively, we examined whether myeloid-associated AhR signaling regulates systemic and tumor immunity. Genetic depletion of AhR in these lineages induced systemic immune activation, characterized by increased IFN-γ production and broad peripheral T-cell activation. This immune activation was propagated into the tumor microenvironment, where AhR loss reduced Tregs and PMN-MDSCs while increasing intratumoral CD8⁺ T-cell infiltration and effector-memory differentiation. These data suggest that myeloid-intrinsic AhR signaling contributes to immune suppression and that its disruption promotes systemic-to-local antitumor immune reprogramming.

Consistent with these findings, pharmacologic AhR inhibition with DA-4505 enhanced antitumor immunity in immune checkpoint inhibitor-resistant models. In the YHIM-2004 LUSC model, which was poorly responsive to pembrolizumab monotherapy, DA-4505 combined with pembrolizumab significantly improved tumor control. Single-cell RNA sequencing revealed enhanced CD8⁺ T-cell cytotoxicity, IFN-γ signaling, and tumor-killing programs, indicating that AhR inhibition can restore T-cell effector function and sensitize resistant tumors to PD-1 blockade.

Mechanistically, AhR inhibition reshaped myeloid-cell states within the tumor microenvironment. IL6⁺ macrophages showed reduced Treg-recruiting capacity, whereas macrophages and dendritic cells displayed enhanced antigen-presentation and T-cell–recruiting programs. Increased MHC-I expression in myeloid cells and strengthened myeloid–CD8⁺ T-cell interactions support a model in which AhR inhibition promotes productive myeloid–T-cell crosstalk. These findings suggest that AhR blockade does not simply increase immune-cell infiltration but functionally redirects the immune network toward T-cell activation and antitumor effector differentiation. As previously reported, elevated IL-6 levels across tumor types are associated with poor ICI responses; mechanistically, IL-6/STAT3 signaling represses cytotoxic differentiation of CD8⁺ T cells, yielding functionally blunted CTLs in patients with high plasma IL-6 levels [42]. Consistent with these reports, preclinical studies have shown that IL-6 or IL-6R blockade synergizes with anti–PD-1/PD-L1 to regress established tumors, increase intratumoral effector CD8⁺ T cells, remodel stroma, and prolong survival [43, 44].

The therapeutic relevance of this mechanism was further supported across multiple tumor contexts. In the ICI-resistant LLC1 model, AhR inhibition combined with anti–PD-1 enhanced CD8⁺ T-cell proliferation and IFN-γ signaling. In the TC-1 lung cancer model, AhR inhibition potentiated both immune checkpoint blockade and chemotherapy, suggesting that AhR blockade may amplify therapy-induced immune priming [45]. In the 4T1 TNBC model, DA-4505 increased immune infiltration, reduced metastatic burden, and improved survival, including in the post-surgical setting [46]. In the colorectal cancer model, AhR inhibition also promoted T cell–dependent tumor control through macrophage–T-cell interactions. Together, these results indicate that AhR inhibition enhances antitumor immunity across diverse tumor microenvironments by promoting T-cell activation, while the contribution of myeloid/APC remodeling varies depending on the tumor immune context.

Differences in depletion outcomes between LLC1 and TC-1 likely reflect distinct baseline immune architectures. The ICI-refractory LLC1 model exhibited a macrophage-rich, CD8⁺ T-cell-low TME, whereas the ICI-sensitive TC-1 model showed greater CD8⁺ T-cell infiltration (Figs. 3E and 4C). Because tumor models differ in the abundance and functional dominance of suppressive immune populations, including TAMs, MDSCs, and Tregs, the relative contribution of each compartment to therapeutic response may vary across tumor contexts [47–49]. Accordingly, macrophage depletion more substantially attenuated therapeutic efficacy in LLC1, whereas CD8⁺ T-cell depletion more strongly reversed tumor control in TC-1. These findings suggest that AhR inhibition may converge on enhanced antitumor T-cell immunity through context-dependent upstream mechanisms, including myeloid/APC remodeling, relief of suppressive myeloid or regulatory T-cell programs, and direct enhancement of effector T-cell activation. Although our mechanistic analyses primarily focused on myeloid–T-cell crosstalk, NK cells and other suppressive immune populations may also contribute to AhR blockade-associated antitumor immunity in a context-dependent manner. In our immune profiling and depletion experiments, NK-cell and MDSC-related changes were observed in selected models but were not consistently detected across all tumor contexts. Therefore, we focused our main mechanistic interpretation on myeloid/APC remodeling and T-cell activation, which were more consistently observed across the genetic and pharmacologic models. Nevertheless, NK cells are key innate effector cells in the TME and can promote tumor control through direct cytotoxicity, cytokine production, and interactions with myeloid and T-cell compartments [50]. Recent studies have highlighted the therapeutic relevance of NK cell–based cancer immunotherapy and reported that NK cell transfer can overcome resistance to PD-(L)1 therapy by remodeling the TME [51]. Thus, the antitumor effects observed after AhR deletion or inhibition should not be interpreted as being mediated exclusively by macrophage–T-cell interactions, but may instead reflect coordinated immune remodeling involving macrophages/dendritic cells, T cells, NK cells, and suppressive myeloid populations.

Several limitations should be noted. Although our study provides comprehensive immune profiling across multiple tumor models, the causal link between AhR inhibition, IL6⁺ macrophage remodeling, and Treg recruitment was not directly tested using cytokine blockade, pathway inhibition, or macrophage-specific perturbation. Future studies targeting IL-6–STAT3, NF-κB signaling, and myeloid-specific AhR-dependent transcriptional programs will be needed to define the precise mechanisms underlying AhR-mediated immune regulation. In addition, validation using patient-derived samples and biomarker analyses will be important to identify tumor contexts most likely to benefit from AhR inhibition. Lastly, although our study focused primarily on myeloid–T-cell crosstalk, our findings do not exclude a contribution of NK cells to AhR blockade-associated antitumor immunity. Because AhR may affect multiple immune compartments, additional studies are warranted to determine whether AhR deletion or inhibition alters NK-cell activation, NK-cell cytotoxicity, or coordinated NK–myeloid–T-cell crosstalk within the tumor microenvironment. Another limitation is that several in vivo experiments included unequal sample sizes across treatment groups due to model-related feasibility constraints and experimental attrition. In particular, the humanized PDX experiment included a small number of evaluable animals in some groups, including the COMBI group. Therefore, the tumor growth and immune remodeling findings from this model should be interpreted as preliminary and supportive rather than definitive evidence of therapeutic synergy or translational efficacy. Although statistical analyses were performed using individual mice as biological replicates, findings from experiments with smaller group sizes should be interpreted with appropriate caution.

In conclusion, our study demonstrates that AhR inhibition reprograms systemic and local antitumor immunity by enhancing myeloid antigen presentation, strengthening myeloid–T-cell crosstalk, and promoting CD8⁺ T-cell activation. These findings provide a mechanistic rationale for developing AhR inhibitors as combination partners for immune checkpoint blockade, chemotherapy, and perioperative therapeutic strategies.

## Conclusion

Using conditional AhR knockout mice, we demonstrated that genetic ablation of AhR enhances systemic immunity, which subsequently augments local antitumor responses. Pharmacologic inhibition of AhR similarly strengthened antitumor immunity, and its combination with clinically used modalities—surgery, chemotherapy, and immunotherapy—resulted in more durable tumor control. Together, these findings suggest that integrating AhR inhibition with standard cancer therapies may provide improved therapeutic benefit for patients.

## Supplementary Information

Below is the link to the electronic supplementary material.

Supplementary Material 1.

## Data Availability

Raw and processed sequencing data from single-cell RNA sequencing and spatial transcriptomic profiling generated in this study are available at https://figshare.com/s/539630820bc2e318ed6c. All original code used for scRNA-seq and spatial transcriptomic analyses has been deposited in GitHub and is publicly available at https://github.com/ytkim97/DA4505_Code/ as of the date of publication. Code used for other analyses in this paper will be made available from the lead contact upon reasonable request.

## Declarations

### Generative AI and AI-assisted technologies

During the preparation of this manuscript, generative AI tools (ChatGPT, OpenAI) were used to improve the clarity and readability of the text. After using this tool, the authors carefully reviewed and edited the content as needed and take full responsibility for the final version of the manuscript. The final English language review and editing were performed by Editage.
