# Supplementary material for: Blocking aryl hydrocarbon receptor remodels myeloid–T cell crosstalk and enhances anti-PD-1 response in the tumor microenvironment
Source: Exp Hematol Oncol. 2026 Oct 26;15:112. doi: 10.1186/s40164-026-00843-8 (PMC13711902; doi:10.1186/s40164-026-00843-8)
Supplement: Supplementary file 1 [file 40164_2026_843_MOESM1_ESM.pdf]

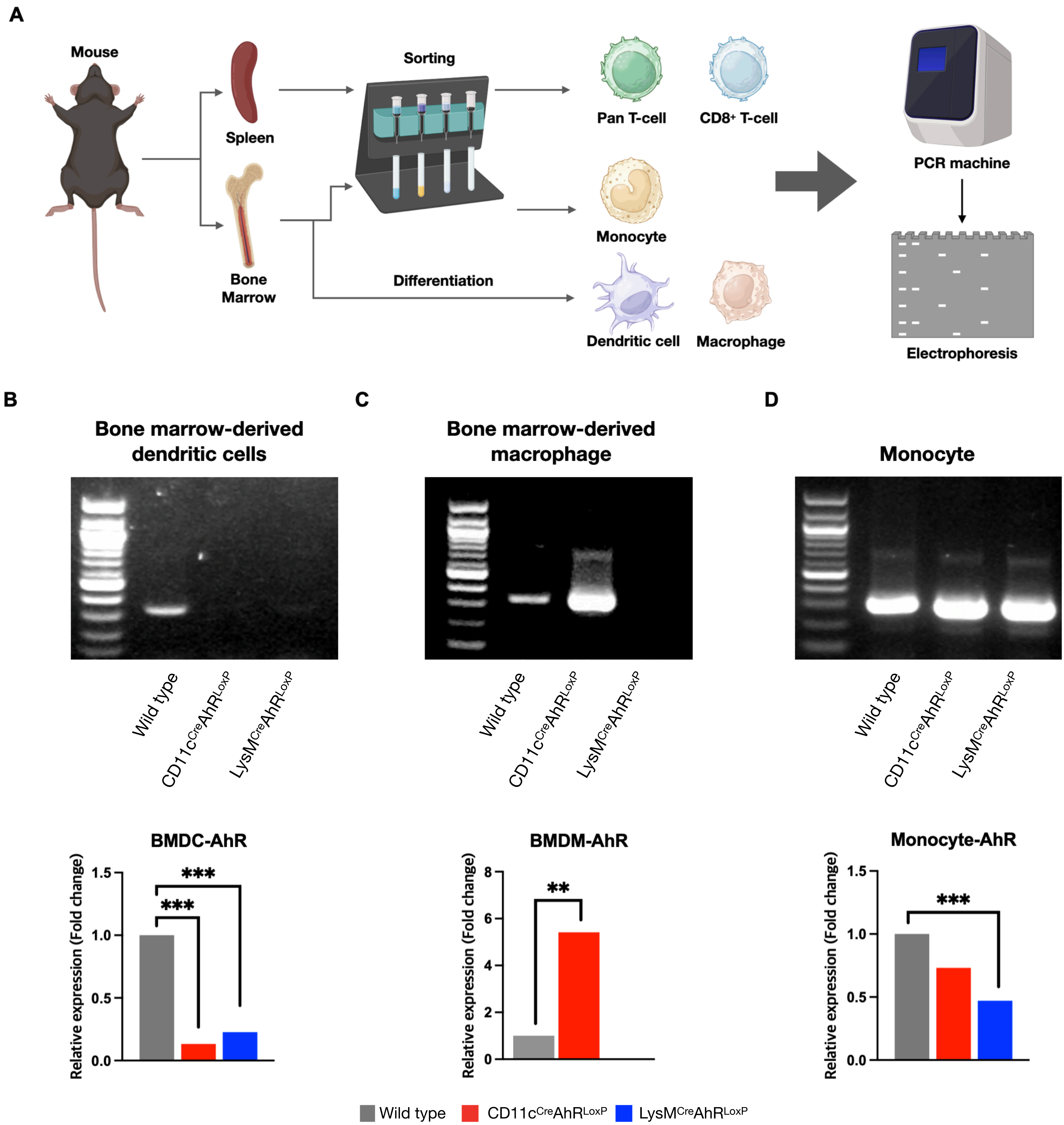

A
Mouse
Spleen
Bone Marrow
Sorting
Differentiation
Pan T-cell
CD8+ T-cell
Monocyte
Dendritic cell
Macrophage
PCR machine
Electrophoresis
B
Bone marrow-derived dendritic cells
C
Bone marrow-derived macrophage
D
Monocyte
Wild type
CD11cCreAhRLoxP
LysMCreAhRLoxP
BMDC-AhR
BMDM-AhR
Monocyte-AhR
Relative expression (Fold change)
***
**

Figure S1. Validation of AhR^{KO} mouse in ex vivo.

(A) Work-flow of the validation design.

(B) AhR gene expression in bone marrow-derived dendritic cells (BMDCs) (n=3 mice per group).

(C) AhR gene expression in bone marrow-derived macrophages (BMDMs) (n=3 mice per group).

(D) AhR gene expression in monocytes. All statistical values of qPCR were calculated by comparing the sample to sample (n=3 mice per group).

Statistical significance between groups was determined using Student's t-test (*P<0.05, **P≤0.01, ***P≤0.001).

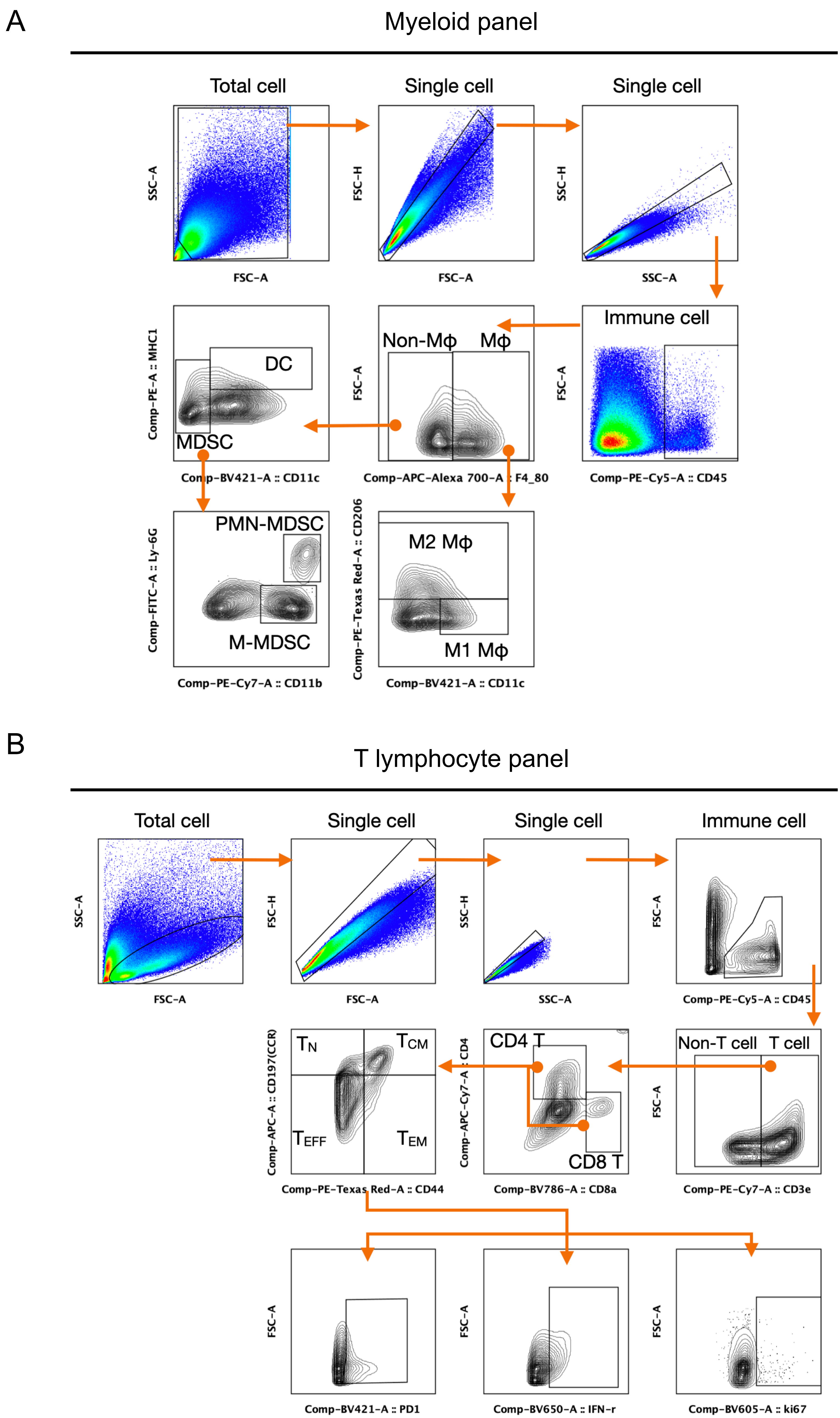
A
Myeloid panel
Total cell
Single cell
Single cell
SSC-A
FSC-A
FSC-H
FSC-A
SSC-H
SSC-A
Immune cell
Non-Mϕ
Mϕ
DC
MDSC
Comp-PE-A :: MHC1
Comp-BV421-A :: CD11c
FSC-A
Comp-APC-Alexa 700-A :: F4_80
FSC-A
Comp-PE-Cy5-A :: CD45
PMN-MDSC
M-MDSC
Comp-FITC-A :: Ly-6G
Comp-PE-Cy7-A :: CD11b
M2 Mϕ
M1 Mϕ
Comp-PE-Texas Red-A :: CD206
Comp-BV421-A :: CD11c
B
T lymphocyte panel
Total cell
Single cell
Single cell
Immune cell
SSC-A
FSC-A
FSC-H
FSC-A
SSC-H
SSC-A
FSC-A
Comp-PE-Cy5-A :: CD45
TN
TCM
TEFF
TEM
Comp-APC-A :: CD197(CCR)
Comp-PE-Texas Red-A :: CD44
CD4 T
CD8 T
Comp-APC-Cy7-A :: CD4
Comp-BV786-A :: CD8a
Non-T cell
T cell
FSC-A
Comp-PE-Cy7-A :: CD3e
FSC-A
Comp-BV421-A :: PD1
FSC-A
Comp-BV650-A :: IFN-r
FSC-A
Comp-BV605-A :: ki67

Figure S2. Gating strategy for flow cytometry analysis. The definition of each subset is shown in the figure.

(A) Gating strategy of myeloid panel

(B) Gating strategy of T-lymphocyte panel

A

B

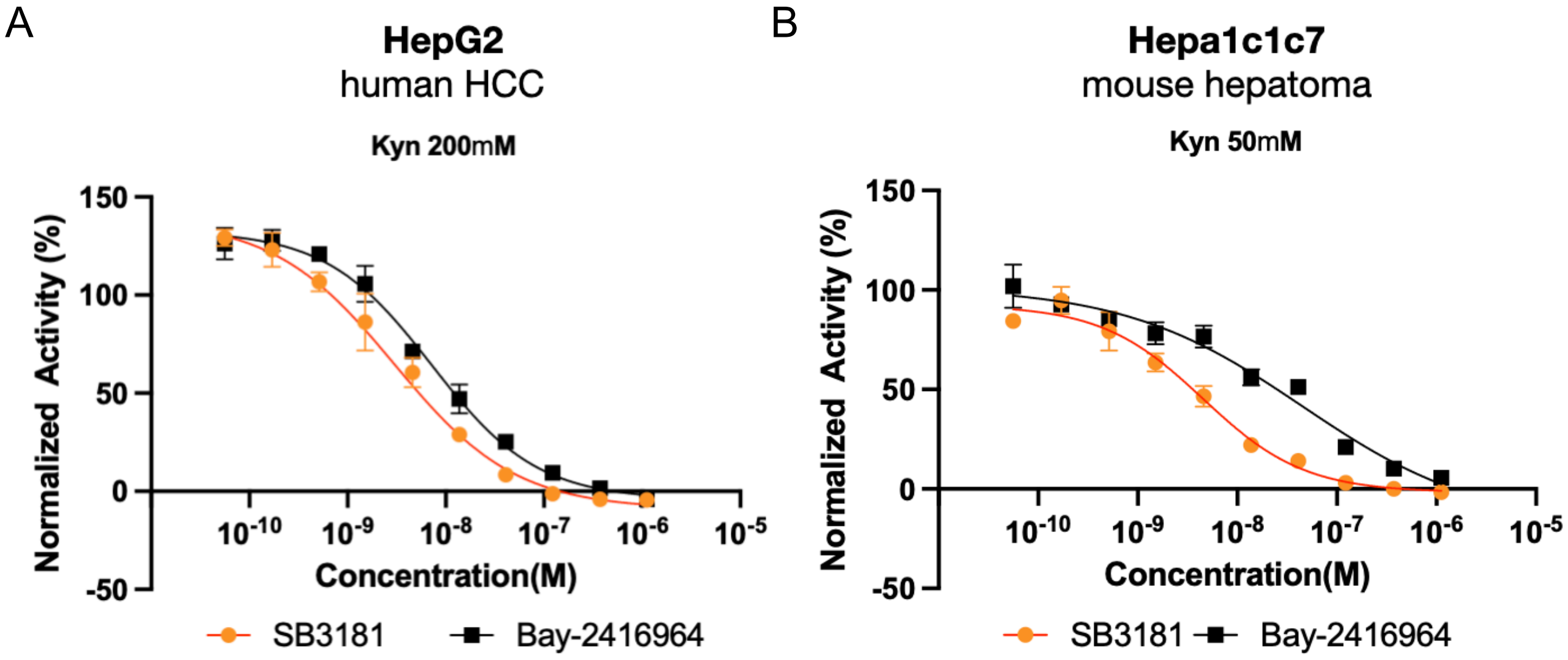

HepG2	SB3181	Bay-2416964
IC_{50} (nM)	2.996	7.210

Hepa1c1c7	SB3181	Bay-2416964
IC_{50} (nM)	4.514	45.003

C

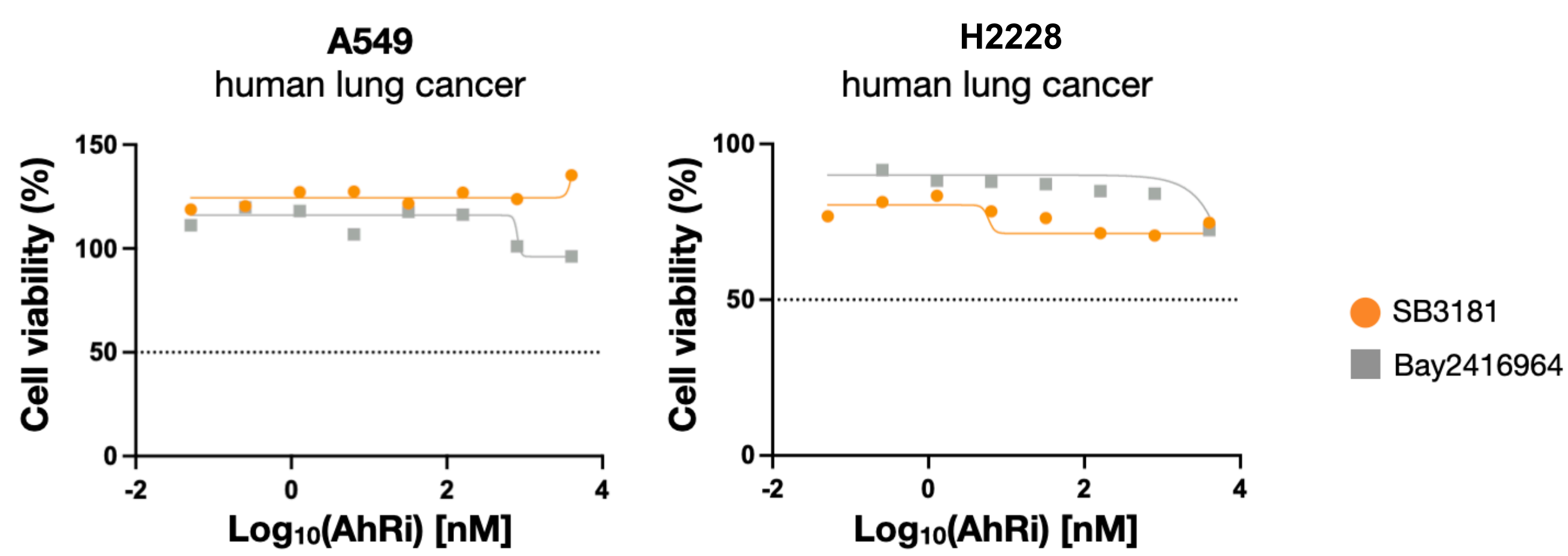

D

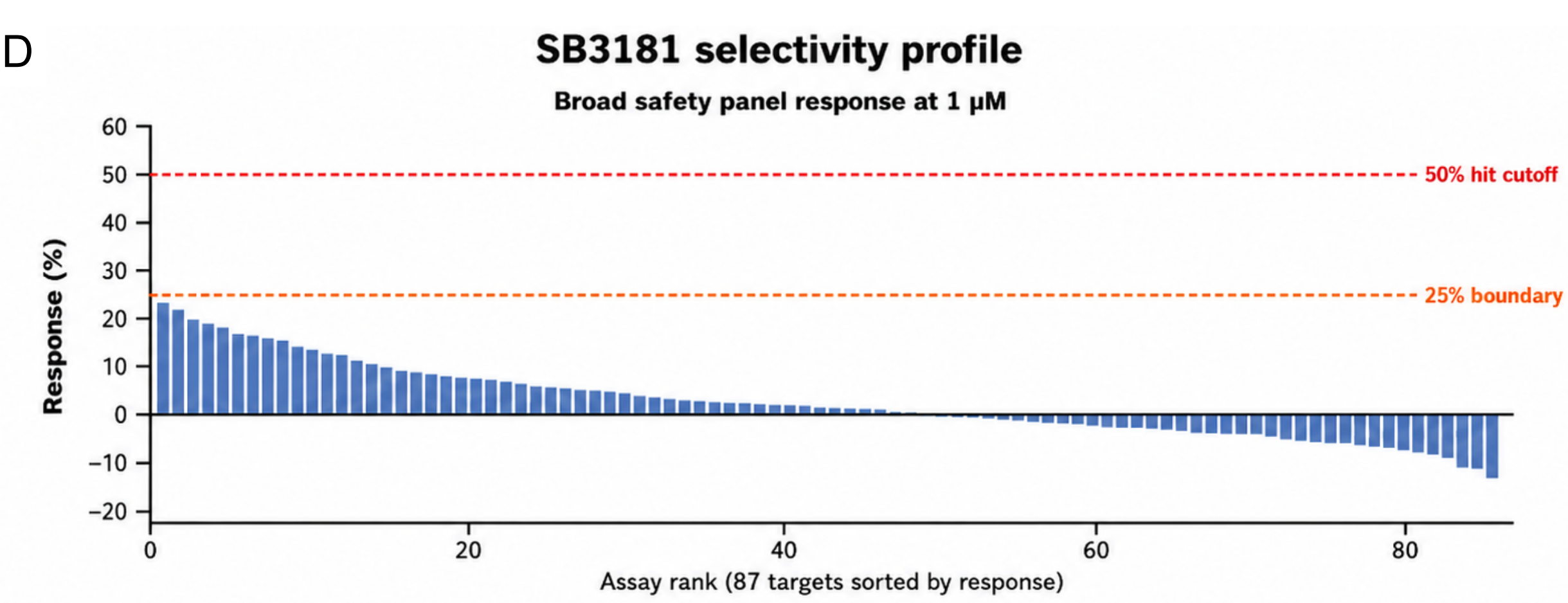

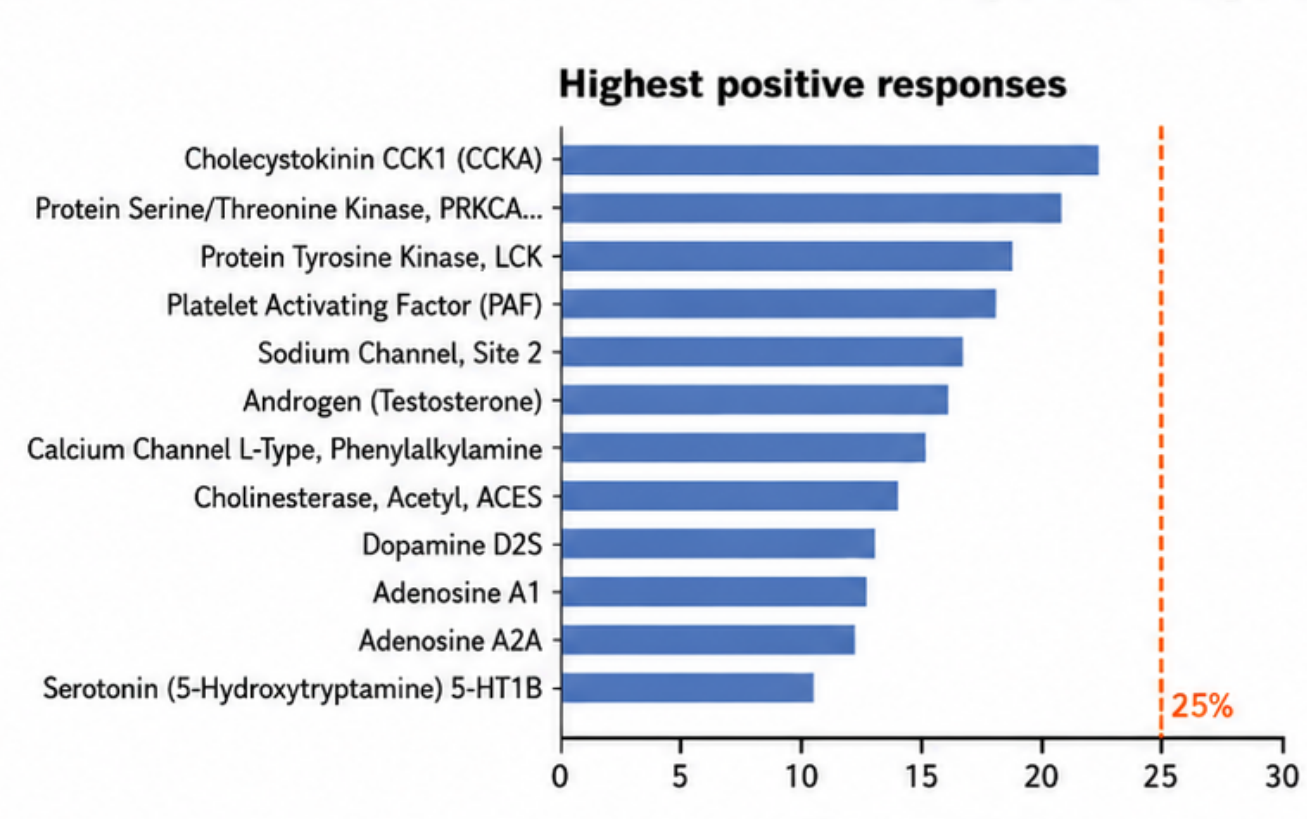

Selectivity summary

Readout	Result
Targets in safety panel	87
Max positive response	23.4%
Targets ≥25% response	0/87
Targets ≥50% response	0/87
PXR antagonist	SB3181: N.C.
TRPV1 agonist	SB3181: N.C.

N.C., IC50 not calculable; concentration-response curve shows <25% effect at the highest validated testing concentration.

Figure S3. SB3181 potently and selectively inhibits AhR signaling with minimal direct cytotoxicity.

(A, B) Inhibition of kynurenine-induced AhR transcriptional activity by SB3181, the active form of DA-4505, and the reference AhR inhibitor BAY 2416964 in HepG2 (human) cells and Hepa1c1c7 (mouse) cells. SB3181 showed lower IC_{50} values than Bay-2416964 in both cell lines.; Kyn, kynurenine; K.A, kynurenine acid

(C) The direct cell-killing effect was not observed by SB3181 treatment in A549 and H2228 cell lines.

(D) Selectivity profile of SB3181 in a broad safety panel. In an 87-target safety screen at 1 μM, SB3181 showed minimal off-target activity, with no targets exceeding the 25% response boundary and no targets meeting the 50% hit cutoff. Follow-up assays against PXR and TRPV1 showed no calculable IC_{50} values, supporting the selective activity profile of SB3181.

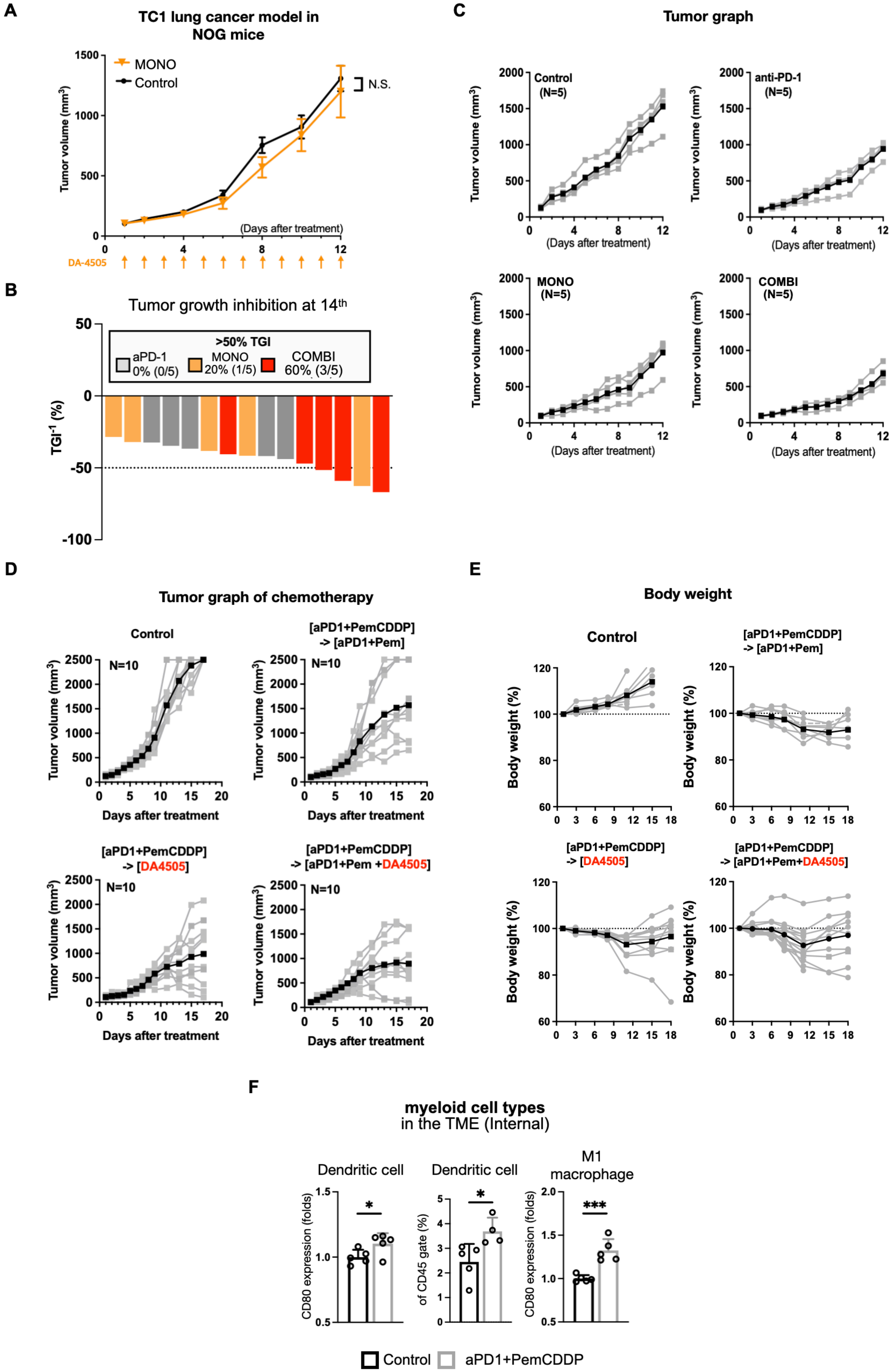

A
TC1 lung cancer model in NOG mice
MONO
Control
N.S.
Tumor volume (mm³)
(Days after treatment)
DA-4505
B
Tumor growth inhibition at 14th
>50% TGI
aPD-1 0% (0/5)
MONO 20% (1/5)
COMBI 60% (3/5)
TGI⁻¹ (%)
C
Tumor graph
Control (N=5)
anti-PD-1 (N=5)
MONO (N=5)
COMBI (N=5)
Tumor volume (mm³)
(Days after treatment)
D
Tumor graph of chemotherapy
Control
[aPD1+PemCDDP] -> [aPD1+Pem]
[aPD1+PemCDDP] -> [DA4505]
[aPD1+PemCDDP] -> [aPD1+Pem +DA4505]
N=10
Tumor volume (mm³)
Days after treatment
E
Body weight
Control
[aPD1+PemCDDP] -> [aPD1+Pem]
[aPD1+PemCDDP] -> [DA4505]
[aPD1+PemCDDP] -> [aPD1+Pem+DA4505]
Body weight (%)
F
myeloid cell types
in the TME (Internal)
Dendritic cell
Dendritic cell
M1 macrophage
CD80 expression (folds)
of CD45 gate (%)
CD80 expression (folds)
*
*
***
Control
aPD1+PemCDDP

Figure S4. Anti-tumor effects were consistently observed in vivo without variation.

(A) There was no tumor regression when DA-4505 was administered (n = 5 mice per group).

(B) Tumor growth inhibition graph (TGI) (n = 5 mice per group).

(C) Individual tumor size graph.

(D) Individual tumor size graph of chemotherapy mouse model (n = 10 mice per group).

(E) Individual body weights of chemotherapy mouse model.

(F) Flow cytometric analysis of dendritic cells and M1 macrophages at the interim time point in the control (n = 5) and anti–PD-1 plus pemetrexed/cisplatin (n = 4–5) groups. CD80 expression in dendritic cells and M1 macrophages and the frequency of dendritic cells were assessed.

Statistical significance between groups was determined using Student's t-test (*$P<0.05$, **$P\leq0.01$, ***$P\leq0.001$).

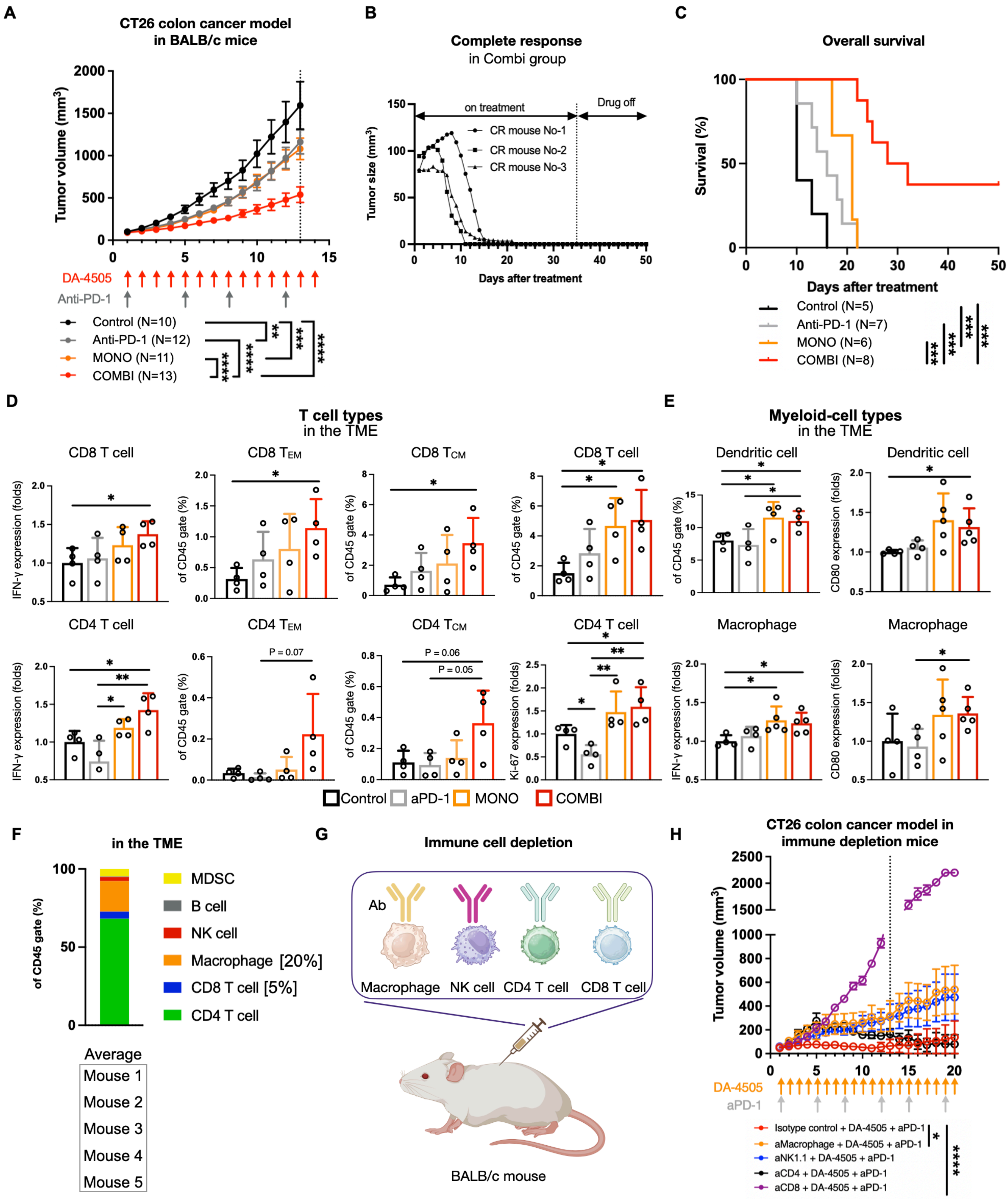
A
CT26 colon cancer model in BALB/c mice
Tumor volume (mm3)
DA-4505
Anti-PD-1
Control (N=10)
Anti-PD-1 (N=12)
MONO (N=11)
COMBI (N=13)
B
Complete response in Combi group
Tumor size (mm3)
on treatment
Drug off
CR mouse No-1
CR mouse No-2
CR mouse No-3
Days after treatment
C
Overall survival
Survival (%)
Days after treatment
Control (N=5)
Anti-PD-1 (N=7)
MONO (N=6)
COMBI (N=8)
D
T cell types in the TME
CD8 T cell
CD8 TEM
CD8 TCM
CD8 T cell
CD4 T cell
CD4 TEM
CD4 TCM
CD4 T cell
IFN-γ expression (folds)
of CD45 gate (%)
Ki-67 expression (folds)
P = 0.07
P = 0.06
P = 0.05
E
Myeloid-cell types in the TME
Dendritic cell
Dendritic cell
Macrophage
Macrophage
CD80 expression (folds)
IFN-γ expression (folds)
Control
aPD-1
MONO
COMBI
F
in the TME
of CD45 gate (%)
MDSC
B cell
NK cell
Macrophage [20%]
CD8 T cell [5%]
CD4 T cell
Average
Mouse 1
Mouse 2
Mouse 3
Mouse 4
Mouse 5
G
Immune cell depletion
Ab
Macrophage
NK cell
CD4 T cell
CD8 T cell
BALB/c mouse
H
CT26 colon cancer model in immune depletion mice
Tumor volume (mm3)
DA-4505
aPD-1
Isotype control + DA-4505 + aPD-1
aMacrophage + DA-4505 + aPD-1
aNK1.1 + DA-4505 + aPD-1
aCD4 + DA-4505 + aPD-1
aCD8 + DA-4505 + aPD-1

Figure S5. Inhibition of AhR enhanced T cell activation and myeloid cell function, leading to robust anti-tumor effects in the CT-26 model.

(A) Tumor growth in the CT26 colon cancer model in BALB/c mice treated with control (n = 10), anti–PD-1 (n = 12), MONO (n = 11), or COMBI (n = 13).

(B) Individual tumor growth curves of mice achieving complete responses in the COMBI group during treatment and following drug withdrawal.

(C) Overall survival of CT26 tumor-bearing mice in the control (n = 5), anti–PD-1 (n = 7), MONO (n = 6), and COMBI (n = 8) groups.

(D) Flow cytometric analysis of tumor-infiltrating T-cell populations following control, anti–PD-1, MONO, or COMBI treatment. IFN-γ expression in $CD8^+$ and $CD4^+$ T cells, frequencies of $CD8^+$ and $CD4^+$ T_{EM} and T_{CM} subsets, total $CD8^+$ T-cell frequency, and Ki-67 expression in $CD4^+$ T cells were assessed.

(E) Flow cytometric analysis of tumor-infiltrating myeloid cell populations following control, anti–PD-1, MONO, or COMBI treatment. Dendritic cell frequency and CD80 expression, together with IFN-γ and CD80 expression in macrophages, were assessed.

(F) Composition of tumor-infiltrating immune cell populations in the CT26 tumor microenvironment (n = 5 mice).

(G) Schematic representation of the immune cell depletion experiment targeting macrophages, NK cells, $CD4^+$ T cells, or $CD8^+$ T cells in BALB/c mice.

(H) Tumor growth in the CT26 colon cancer model following immune cell depletion in mice treated with DA-4505 and anti–PD-1. Groups included isotype control (n = 4), macrophage-depleted (n = 5), NK cell-depleted (n = 5), $CD4^+$ T-cell-depleted (n = 4), and $CD8^+$ T-cell-depleted (n = 5) mice.

Statistical significance among the groups was calculated using Student's t-test ($P<0.05$) and two-way ANOVA followed by Tukey's multiple comparisons test (*$P<0.05$, **$P\leq0.01$, ***$P\leq0.001$, ****$P\leq0.0001$).

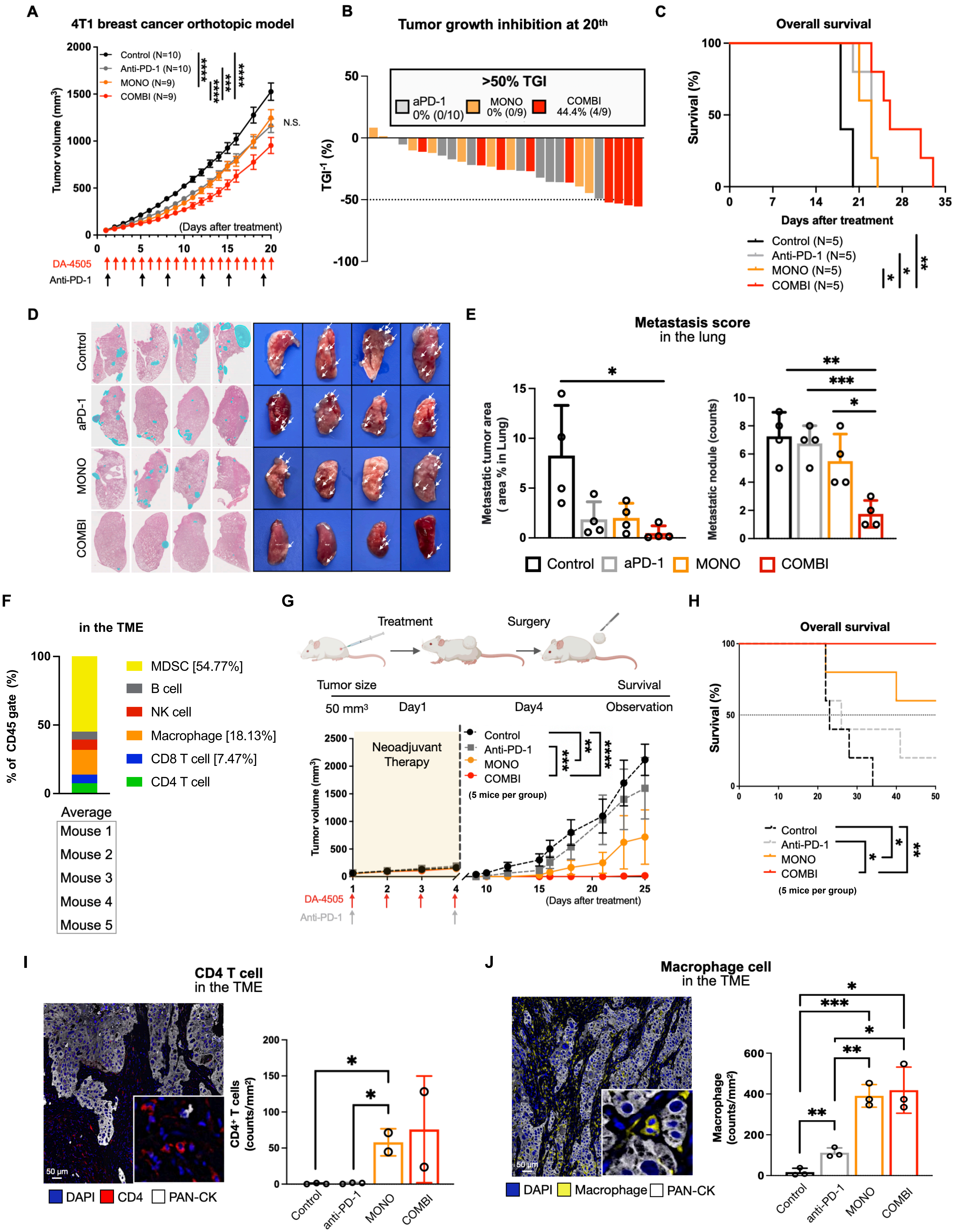

A
4T1 breast cancer orthotopic model
Control (N=10)
Anti-PD-1 (N=10)
MONO (N=9)
COMBI (N=9)
Tumor volume (mm³)
N.S.
(Days after treatment)
DA-4505
Anti-PD-1
B
Tumor growth inhibition at 20th
>50% TGI
aPD-1 0% (0/10)
MONO 0% (0/9)
COMBI 44.4% (4/9)
TGI-1 (%)
C
Overall survival
Survival (%)
Days after treatment
Control (N=5)
Anti-PD-1 (N=5)
MONO (N=5)
COMBI (N=5)
D
Control
aPD-1
MONO
COMBI
E
Metastasis score
in the lung
Metastatic tumor area (area % in Lung)
Metastatic nodule (counts)
Control
aPD-1
MONO
COMBI
F
in the TME
% of CD45 gate (%)
MDSC [54.77%]
B cell
NK cell
Macrophage [18.13%]
CD8 T cell [7.47%]
CD4 T cell
Average
Mouse 1
Mouse 2
Mouse 3
Mouse 4
Mouse 5
G
Treatment
Surgery
Tumor size
Survival
50 mm³
Day1
Day4
Observation
Neoadjuvant Therapy
Control
Anti-PD-1
MONO
COMBI
(5 mice per group)
Tumor volume (mm³)
(Days after treatment)
DA-4505
Anti-PD-1
H
Overall survival
Survival (%)
Control
Anti-PD-1
MONO
COMBI
(5 mice per group)
I
CD4 T cell
in the TME
50 μm
DAPI
CD4
PAN-CK
CD4+ T cells (counts/mm²)
Control
anti-PD-1
MONO
COMBI
J
Macrophage cell
in the TME
50 μm
DAPI
Macrophage
PAN-CK
Macrophage (counts/mm²)
Control
anti-PD-1
MONO
COMBI

Figure S6. AhR inhibition induces macrophage and CD4+ T cell infiltration, enhancing anti-tumor and anti-metastatic effects in the TNBC 4T1 orthotopic model.

(A) Tumor growth in the 4T1 breast cancer orthotopic model in BALB/c mice treated with control (n = 10), anti–PD-1 (n = 10), MONO (n = 9), or COMBI (n = 9).

(B) Tumor growth inhibition (TGI) at day 20 in the anti–PD-1 (n = 10), MONO (n = 9), and COMBI (n = 9) groups.

(C) Overall survival of 4T1 tumor-bearing mice in the control, anti–PD-1, MONO, and COMBI groups (n = 5 mice per group).

(D) Representative H&E-stained lung sections and gross lung images from the control, anti–PD-1, MONO, and COMBI groups. Metastatic tumor regions in H&E sections are highlighted in blue, and metastatic nodules in gross lung specimens are indicated by white arrows.

(E) Quantification of lung metastasis based on metastatic tumor area and the number of metastatic nodules in the control, anti–PD-1, MONO, and COMBI groups.

(F) Composition of tumor-infiltrating immune cell populations in the 4T1 tumor microenvironment (n = 5 mice).

(G) Schematic representation of the neoadjuvant treatment and tumor resection experiment in the 4T1 orthotopic breast cancer model. Mice received control, anti–PD-1, MONO, or COMBI treatment before surgical tumor resection, followed by monitoring of tumor growth after surgery (n = 5 mice per group).

(H) Overall survival following neoadjuvant treatment and surgical tumor resection in the control, anti–PD-1, MONO, and COMBI groups (n = 5 mice per group).

(I) Representative multiplex immunohistochemistry (mIHC) images and quantification of $CD4^+$ T-cell infiltration in 4T1 tumor tissues from the control (n = 3), anti–PD-1 (n = 3), MONO (n = 2), and COMBI (n = 2) groups. DAPI, CD4, and Pan-CK are shown by the indicated markers. Scale bar, 50 μm.

(J) Representative mIHC images and quantification of macrophage infiltration in 4T1 tumor tissues from the control, anti–PD-1, MONO, and COMBI groups (n = 3 mice per group). DAPI, macrophage, and Pan-CK are shown by the indicated markers. Scale bar, 50 μm.

Statistical significance among the groups was calculated using Student's t-test (P<0.05) and two-way ANOVA followed by Tukey's multiple comparisons test (*P<0.05, **P≤0.01, ***P≤0.001, ****P≤0.0001).; Bar size = 50 μm; Blue color, DAPI; Red color, CD4 T cell; Yellow color, Macrophage; White color, Cancer cell.

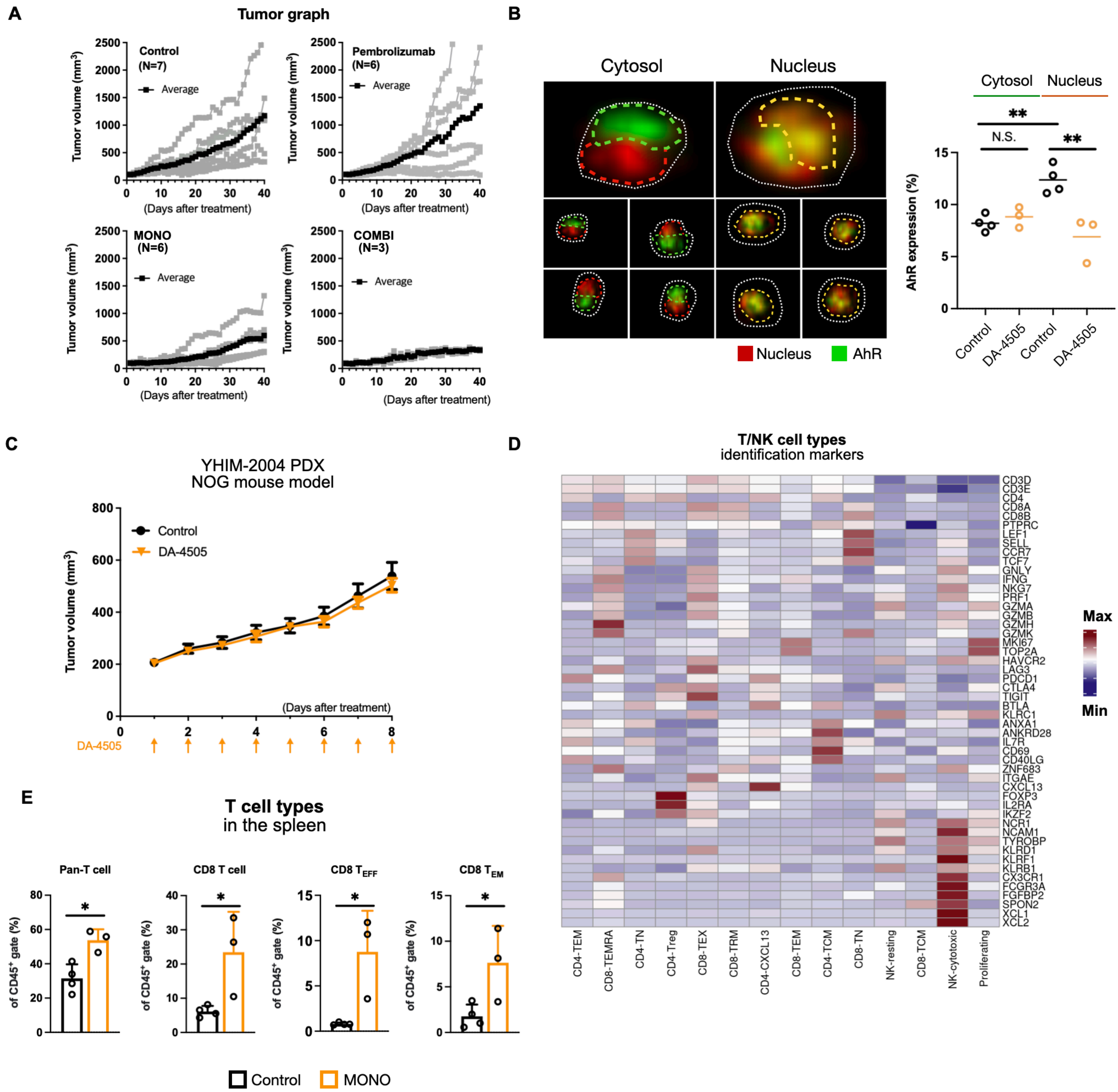
A
Tumor graph
Control
(N=7)
Average
Pembrolizumab
(N=6)
MONO
(N=6)
COMBI
(N=3)
Tumor volume (mm³)
(Days after treatment)
B
Cytosol
Nucleus
Nucleus
AhR
Cytosol
Nucleus
**
N.S.
**
AhR expression (%)
Control
DA-4505
Control
DA-4505
C
YHIM-2004 PDX
NOG mouse model
Control
DA-4505
Tumor volume (mm³)
(Days after treatment)
DA-4505
D
T/NK cell types
identification markers
CD3D
CD3E
CD4
CD8A
CD8B
PTPRC
LEF1
SELL
CCR7
TCF7
GNLY
IFNG
NKG7
PRF1
GZMA
GZMB
GZMH
GZMK
MKI67
TOP2A
HAVCR2
LAG3
PDCD1
CTLA4
TIGIT
BTLA
KLRC1
ANXA1
ANKRD28
IL7R
CD69
CD40LG
ZNF683
ITGAE
CXCL13
FOXP3
IL2RA
IKZF2
NCR1
NCAM1
TYROBP
KLRD1
KLRF1
KLRB1
CX3CR1
FCGR3A
FGFBP2
SPON2
XCL1
XCL2
Max
Min
CD4-TEM
CD8-TEMRA
CD4-TN
CD4-Treg
CD8-TEX
CD8-TRM
CD4-CXCL13
CD8-TEM
CD4-TCM
CD8-TN
NK-resting
CD8-TCM
NK-cytotoxic
Proliferating
E
T cell types
in the spleen
Pan-T cell
CD8 T cell
CD8 T_EFF
CD8 T_EM
of CD45⁺ gate (%)
Control
MONO

Figure S7. AhR is effectively inhibited by DA-4505.

(A) Individual tumor growth curves in the YHIM-2004 patient-derived xenograft (PDX) model treated with control (n = 7), pembrolizumab (n = 6), MONO (n = 6), or COMBI (n = 3). Black lines indicate the mean tumor volume for each group.

(B) Representative fluorescence images and quantification of cytosolic and nuclear AhR localization in the control (n = 4) and DA-4505-treated (n = 3) groups. AhR and nuclei are shown in green and red, respectively.

(C) Tumor growth in the YHIM-2004 PDX NOG mouse model treated with control (n = 9) or DA-4505 (n = 7).

(D) Heatmap showing the expression of canonical identification markers used to characterize T- and NK-cell subtypes identified by scRNA-seq.

(E) Flow cytometric analysis of splenic T-cell populations in the control (n = 4) and MONO (n = 3) groups. The frequencies of pan-T cells, $CD8^+$ T cells, $CD8^+$ T_{EFF} cells, and $CD8^+$ T_{EM} cells within the $CD45^+$ population were assessed.

All statistical values of quantification were calculated by comparing the sample to sample. Statistical significance among samples was determined using Student’s t-test (*$P<0.05$, **$P\leq0.01$, ***$P\leq0.001$).

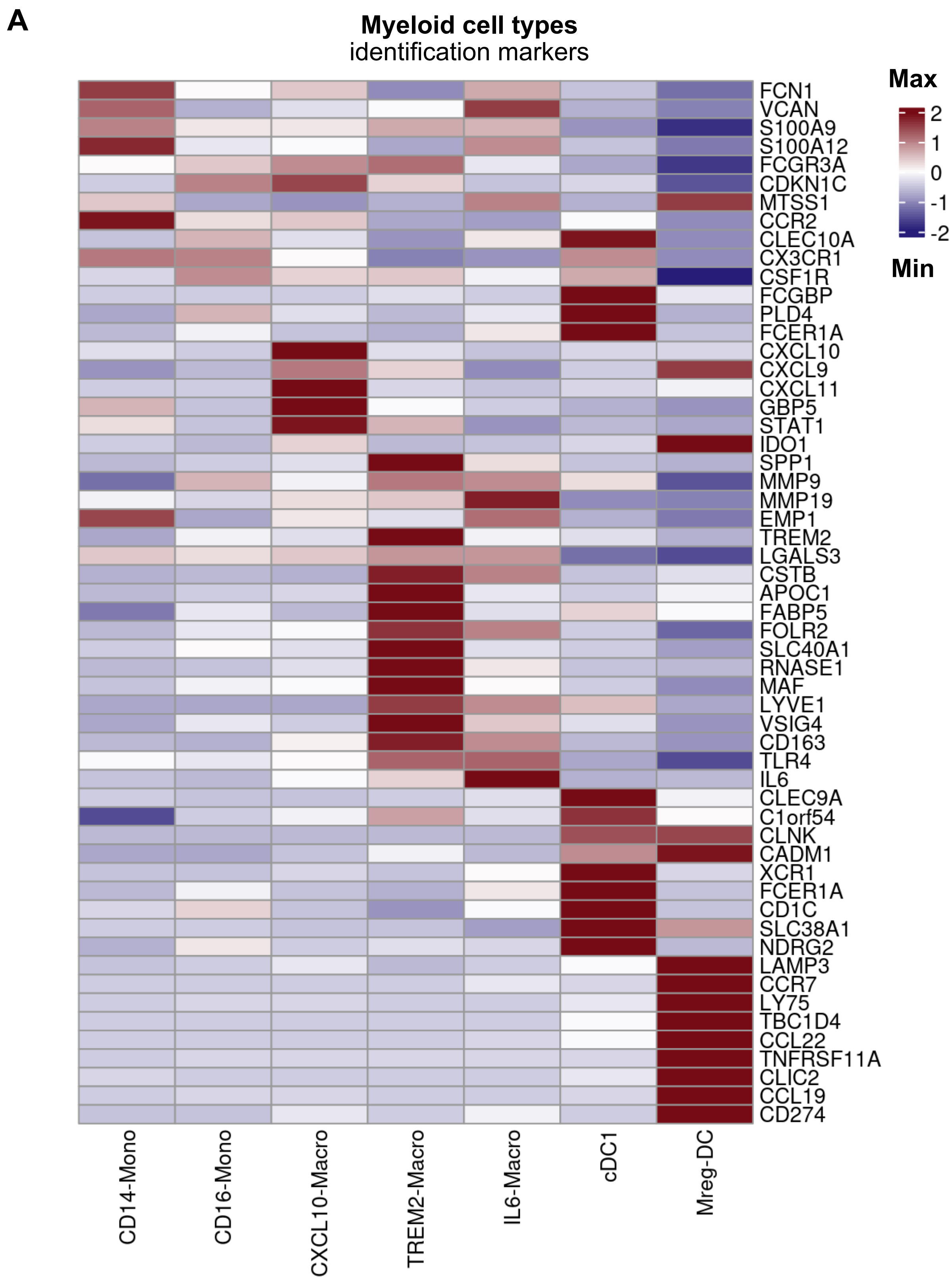
A
Myeloid cell types
identification markers
Max
2
1
0
-1
-2
Min
FCN1
VCAN
S100A9
S100A12
FCGR3A
CDKN1C
MTSS1
CCR2
CLEC10A
CX3CR1
CSF1R
FCGBP
PLD4
FCER1A
CXCL10
CXCL9
CXCL11
GBP5
STAT1
IDO1
SPP1
MMP9
MMP19
EMP1
TREM2
LGALS3
CSTB
APOC1
FABP5
FOLR2
SLC40A1
RNASE1
MAF
LYVE1
VSIG4
CD163
TLR4
IL6
CLEC9A
C1orf54
CLNK
CADM1
XCR1
FCER1A
CD1C
SLC38A1
NDRG2
LAMP3
CCR7
LY75
TBC1D4
CCL22
TNFRSF11A
CLIC2
CCL19
CD274
CD14-Mono
CD16-Mono
CXCL10-Macro
TREM2-Macro
IL6-Macro
cDC1
Mreg-DC

Figure S8. Identification and annotation of myeloid sub-populations using scRNAseq.

(A) Identification markers of myeloid cell types.

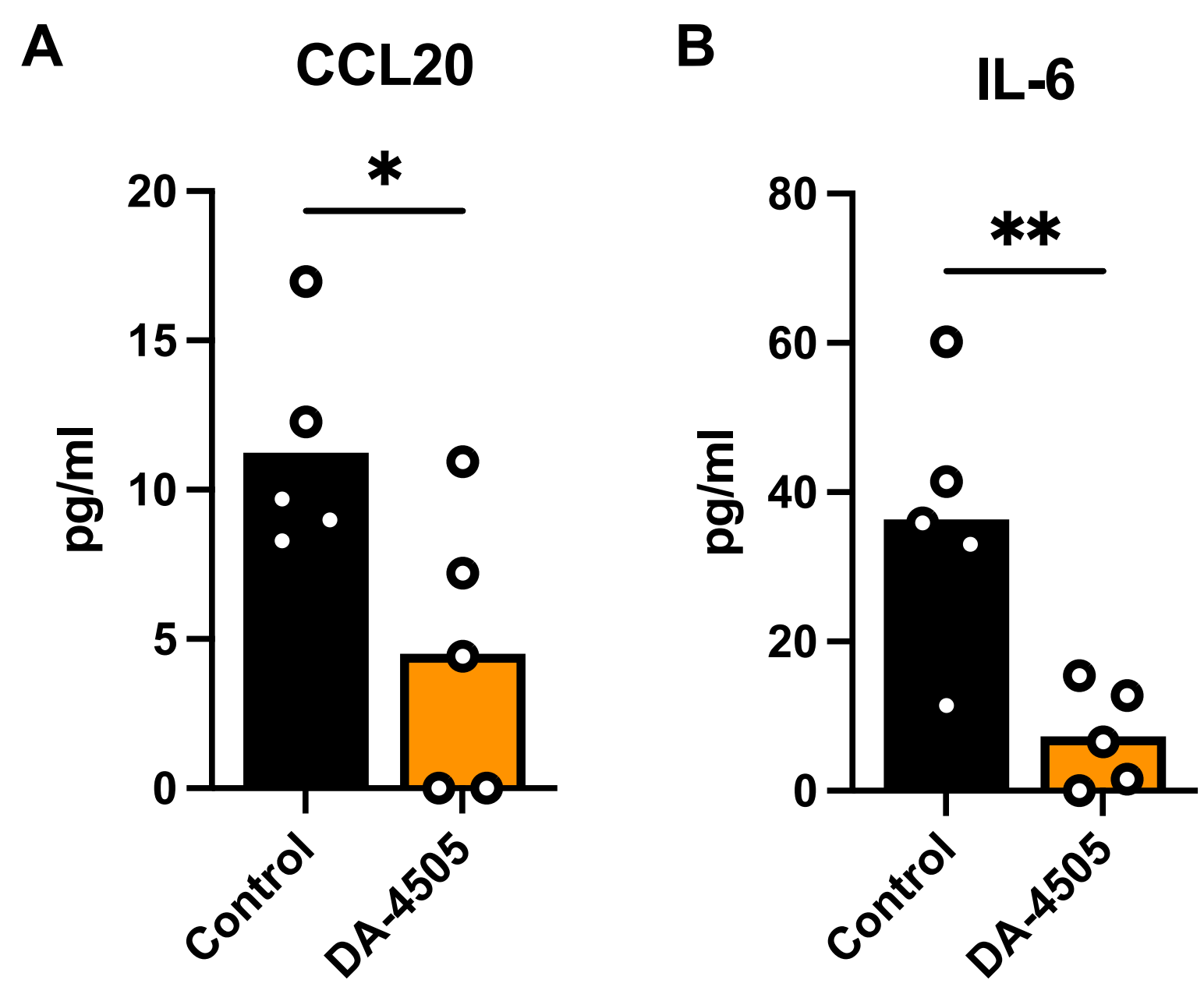
A
CCL20
*
20
15
10
5
0
pg/ml
Control
DA-4505
B
IL-6
**
80
60
40
20
0
pg/ml
Control
DA-4505

Figure S9. Pharmacologic AhR inhibition reduces plasma CCL20 and IL-6 levels in a humanized PDX model.

(A, B) Plasma concentrations of (A) CCL20 and (B) IL-6 in vehicle-treated control and DA-4505-treated humanized PDX-bearing mice, as determined by ELISA (n=5 mice per group).

Each circle represents an individual mouse, and bars indicate the mean. Statistical significance was assessed using a Student's t-test. *P < 0.05; **P < 0.01.

A

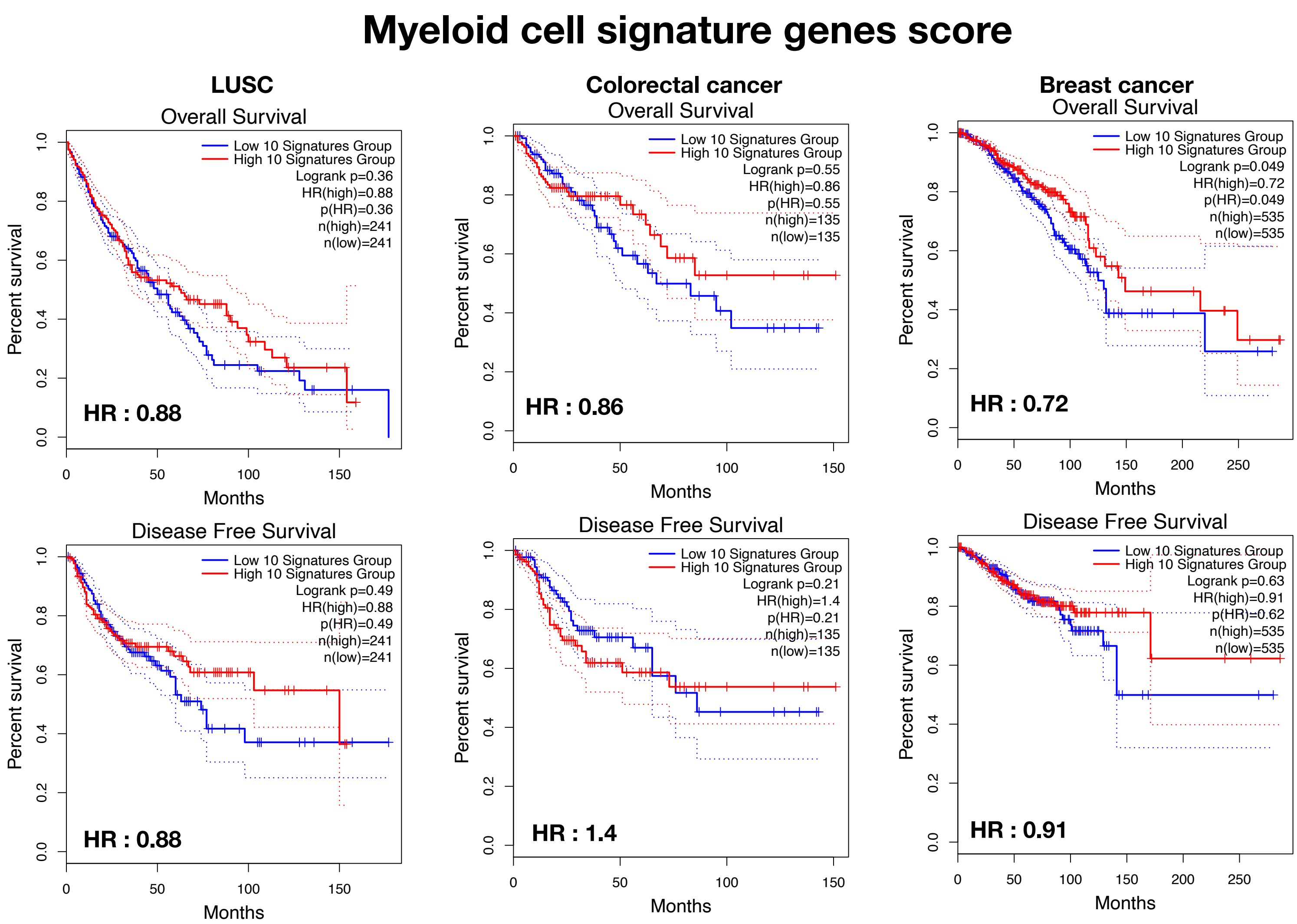

Myeloid cell signature genes score
LUSC
Overall Survival
Low 10 Signatures Group
High 10 Signatures Group
Logrank p=0.36
HR(high)=0.88
p(HR)=0.36
n(high)=241
n(low)=241
HR : 0.88
Colorectal cancer
Overall Survival
Logrank p=0.55
HR(high)=0.86
p(HR)=0.55
n(high)=135
n(low)=135
HR : 0.86
Breast cancer
Overall Survival
Logrank p=0.049
HR(high)=0.72
p(HR)=0.049
n(high)=535
n(low)=535
HR : 0.72
Disease Free Survival
Logrank p=0.49
HR(high)=0.88
p(HR)=0.49
n(high)=241
n(low)=241
HR : 0.88
Disease Free Survival
Logrank p=0.21
HR(high)=1.4
p(HR)=0.21
n(high)=135
n(low)=135
HR : 1.4
Disease Free Survival
Logrank p=0.63
HR(high)=0.91
p(HR)=0.62
n(high)=535
n(low)=535
HR : 0.91
Percent survival
Months

Figure S10. Prognostic relevance of myeloid cell signature gene scores across cancer types in TCGA cohorts.

(A) Kaplan–Meier analysis of overall survival (OS, top panels) and disease-free survival (DFS, bottom panels) in patients stratified by high versus low myeloid cell signature gene scores in TCGA cohorts. Cancer types analyzed include lung squamous cell carcinoma (LUSC, left), colorectal cancer (middle), and breast cancer (right). Patients were divided into high (red) and low (blue) groups according to the top or bottom decile of signature gene expression. Hazard ratios (HR), log-rank p values, and sample numbers (n) are indicated in each plot. Statistical significance among the groups was calculated using the log-rank test for survival analyses.

Cell types	Markers
CD8 TN (naïve)	"CCR7", "SELL", "TCF7", "LEF1"
CD8 T_{CM} (Central memory)	"SELL", "TCF7", "ANXA1", IL7R"
CD8 T_{EM} (Effector memory)	"IL7R", "CD40LG", "ANXA1"
CD8 T_{RM} (Residential memory)	"ZNF683", "ITAGE", "GZMB", "NKG7", "GZMK"
CD8 T_{EX} (exhausted)	"GNLY", "IFNG", "NKG7", "PRF1", "HAVCR2", "LAG3", "PDCD1", "CTLA4", "TIGIT"
CD8 T_{EMRA} (CD45RA^{+} effector memory)	"CX3CR1", "GNLY", "IFNG", "NKG7", "PRF1"
CD4 TN (naïve)	"CCR7", "SELL", "TCF7", "LEF1"
CD4 T_{CM} (Central memory)	"TCF7", "ANXA1", IL7R", “CD40LG”
CD4 T_{EM} (Effector memory)	“PDCD1”, ”ANXA1", IL7R”, “CD40LG”
CXCL13 CD4 T cell	“CXCL13”, “KLRB1”, “CD40LG”, “PDCD1”
CD4 Treg (regulatory)	“CTLA4”, “TIGIT”, “FOXP3”, “IL2RA”

Table S1. Identification markers of eleven T cell phenotype

Cell types	Markers
CD14 monocyte	“FCN1”, “VCAN”, “S100A9”, “S100A12”
CD16 monocyte	“FCGR3A”, “CDKN1C”, “CX3CR1”, “CSF1R”
CXCL10 macrophage	“CXCL10”, “CXCL9”, “CXCL11”, “GBP5”, “STAT1"
TREM2 macrophage	“SPP1”, “MMP9”, “TREM2”, “LGALS3”, “CSTB”
IL6 macrophage	“IL6”, “MMP19”, “EMP1”, “FOLR2”
cDC1	“CLEC9A”, “XCR1”, “FCER1A”, “CD1C”
Mreg DC	“LAMP3”, “CCR7”, “CCL22” ,”CCL19” ,”PD-L1”

Table S2. Identification markers of seven myeloid cell phenotype

**Age**	Total Number (n)	Percentage (%)
41-50	4	11.8
51-60	8	23.5
61-70	14	41.2
71-80	8	23.5
**Sex**	Total Number (n)	Percentage (%)
Female	2	5.9
Male	32	94.1
**Cancer vs Normal**	Total Number (n)	Percentage (%)
Cancer	34	85
**Grade**	Total Number (n)	Percentage (%)
n/a	3	8.8
1	1	2.9
1-2	3	8.8
2	11	32.4
2-3	8	23.5
3	8	23.5
**Stage**	Total Number (n)	Percentage (%)
I	16	47.1
II	14	41.2
III	4	11.8

Table S3. Summary of clinical characteristics of samples included in mIHC analysis

T cell signature genes	Myeloid cell signature genes
“HTRA1”, “PDLIM4”, “LY6D”, “SERINC2”, “PTHLH”, “KRT14”, “GZMH”, “CXCL1”, “FGFBP1”, “LCN2”, “CKB”, “S100A9”, “LAD1”, “SERPINB6”, “KRT5”, “SCX”, “FXYD3”, “PDLIM1”, “FASLG”, “CXCL13”	“S100A7”, “LY6D”, “KRT14”, “FXYD3”, “LCN2”, “KRT6A”, “KRT5”, “FGFBP1”, “KRT17”, “SLPI”

Table S4. T cell and Myeloid cell signature gene set of MONO group (vs control group)

T cell signature genes
“ATP8B4”, “TGFBI”, “TRAV36DV6”, “GPR25”, “LAMB3”, “PTPN13”, “LAMC2”, “SCGB1A1”, “FN1”, “DSP”, “PLS3”, “LAMA3”, “LCN2”, “COL17A1”, “SPRR1B”, “HAVCR2”, “RDH10”, “SERINC2”, “S100A2”, “S100A16”

Table S5. T cell signature gene set of COMBI group (vs pembrolizumab group)
